# *Methylocystis* sp. Strain SC2 Acclimatizes to Increasing NH_4_^+^ Levels by a Precise Rebalancing of Enzymes and Osmolyte Composition

**DOI:** 10.1128/msystems.00403-22

**Published:** 2022-09-26

**Authors:** Kangli Guo, Anna Hakobyan, Timo Glatter, Nicole Paczia, Werner Liesack

**Affiliations:** a Methanotrophic Bacteria and Environmental Genomics/Transcriptomics Research Group, Max Planck Institute for Terrestrial Microbiology, Marburg, Germany; b Core Facility for Mass Spectrometry and Proteomics, Max Planck Institute for Terrestrial Microbiology, Marburg, Germany; c Core Facility for Metabolomics and Small Molecule Mass Spectrometry, Max Planck Institute for Terrestrial Microbiology, Marburg, Germany; d Center for Synthetic Microbiology (SYNMIKRO), Philipps-Universität Marburg, Marburg, Germany; University of California San Diego

**Keywords:** methanotrophs, *Methylocystis*, methane, ammonia, particulate methane monooxygenase, hydroxylamine oxidoreductase, proteomics

## Abstract

A high NH_4_^+^ load is known to inhibit bacterial methane oxidation. This is due to a competition between CH_4_ and NH_3_ for the active site of particulate methane monooxygenase (pMMO), which converts CH_4_ to CH_3_OH. Here, we combined global proteomics with amino acid profiling and nitrogen oxides measurements to elucidate the cellular acclimatization response of *Methylocystis* sp. strain SC2 to high NH_4_^+^ levels. Relative to 1 mM NH_4_^+^, a high (50 mM and 75 mM) NH_4_^+^ load under CH_4_-replete conditions significantly increased the lag phase duration required for proteome adjustment. The number of differentially regulated proteins was highly significantly correlated with an increasing NH_4_^+^ load. The cellular responses to increasing ionic and osmotic stress involved a significant upregulation of stress-responsive proteins, the K^+^ “salt-in” strategy, the synthesis of compatible solutes (glutamate and proline), and the induction of the glutathione metabolism pathway. A significant increase in the apparent *K_m_* value for CH_4_ oxidation during the growth phase was indicative of increased pMMO-based oxidation of NH_3_ to toxic hydroxylamine. The detoxifying activity of hydroxlyamine oxidoreductase (HAO) led to a significant accumulation of NO_2_^−^ and, upon decreasing O_2_ tension, N_2_O. Nitric oxide reductase and hybrid cluster proteins (Hcps) were the candidate enzymes for the production of N_2_O. In summary, strain SC2 has the capacity to precisely rebalance enzymes and osmolyte composition in response to increasing NH_4_^+^ exposure, but the need to simultaneously combat both ionic-osmotic stress and the toxic effects of hydroxylamine may be the reason why its acclimatization capacity is limited to 75 mM NH_4_^+^.

**IMPORTANCE** In addition to reducing CH_4_ emissions from wetlands and landfills, the activity of alphaproteobacterial methane oxidizers of the genus *Methylocystis* contributes to the sink capacity of forest and grassland soils for atmospheric methane. The methane-oxidizing activity of *Methylocystis* spp. is, however, sensitive to high NH_4_^+^ concentrations. This is due to the competition of CH_4_ and NH_3_ for the active site of particulate methane monooxygenase, thereby resulting in the production of toxic hydroxylamine with an increasing NH_4_^+^ load. An understanding of the physiological and molecular response mechanisms of *Methylocystis* spp. is therefore of great importance. Here, we combined global proteomics with amino acid profiling and NOx measurements to disentangle the cellular mechanisms underlying the acclimatization of *Methylocystis* sp. strain SC2 to an increasing NH_4_^+^ load.

## INTRODUCTION

Aerobic methanotrophic bacteria, or methanotrophs, are crucial players in the global cycle of the greenhouse gas methane. These bacteria are defined by their ability to utilize methane as their sole energy source for growth (1). Among the known methane oxidizers, proteobacterial methanotrophs have been unequivocally proven to be functionally important in natural and anthropogenic terrestrial environments (1, 2). Indeed, their activity acts in aerobic interfaces of methanogenic environments as a methane biofilter through which the emission of this greenhouse gas to the atmosphere is greatly mitigated (2–4). Another environmentally relevant activity is their ability to act as a sink for atmospheric CH_4_ in unsaturated soils (5). Their key enzyme is particulate methane monooxygenase (pMMO) (6), which converts CH_4_ to methanol (CH_3_OH). The pMMO is an integral part of an extensive intracytoplasmic membrane system (ICM), which is a particular characteristic of proteobacterial methanotrophs (7, 8).

Historically, these bacteria have been classified into type I and type II methanotrophs. This differentiation was particularly based on the type of ICM, the biochemical pathways of carbon fixation, the capability of nitrogen fixation, the formation of resting stages, and the phospholipid fatty acid composition (9, 10). Phylogenetic analysis of their 16S rRNA gene sequences confirmed the initial classification into type I (*Gammaproteobacteria*) and type II (*Alphaproteobacteria*) methanotrophs. Besides phylogeny, the carbon fixation pathway, however, remained the only major feature of the above-mentioned criteria that validly differentiates between type I and type II methanotrophs. As suggested by Knief (1), we therefore use these terms only as synonyms for the phylogenetic groups of *Gamma*- and *Alphaproteobacteria*. The methanotrophic *Alphaproteobacteria* were further divided into type IIa (*Methylocystaceae*) and type IIb (*Beijerinckiaceae*) methanotrophs (11, 12). Various members of the *Methylocystaceae* are able to produce two pMMO isozymes that exhibit different methane oxidation kinetics (6, 13). These methanotrophs are widely distributed in natural wetlands and rice paddies but have also been shown to be abundantly present in upland (e.g., forest) and grasslands soils, where they may oxidize atmospheric CH_4_ (14–16). Indeed, recent research has unambiguously shown that *Methylocystis* spp. contribute via the expression of their high-affinity pMMO to the atmospheric CH_4_ sink in grasslands, in addition to USCα and USCγ (17).

Like all microorganisms, methanotrophs require nitrogen for growth. Most of them utilize either NO_3_^−^ or NH_4_^+^ as a nitrogen source for growth. The structural homology between pMMO and ammonia monooxygenase, however, allows both methanotrophs and ammonia oxidizers to convert either substrate (CH_4_ or NH_3_), although neither is able to grow on the alternative substrate (18–20). The pMMO oxidizes NH_3_ to hydroxylamine (NH_2_OH) (19). Ammonia produced from the deprotonation of liquid NH_4_^+^ competes with CH_4_ for the same active site of pMMO (21, 22). Whether NH_4_^+^ in the environment has inhibitory or stimulatory effects on methane-oxidizing bacteria depends largely on the diversity, structure, and activity of the methanotrophic community, as well as the particular conditions in the habitat (23–25). Additional information on the impact of NH_4_^+^ on methanotroph ecology can be found in Text S1 in the supplemental material.

10.1128/msystems.00403-22.1 TEXT S1 Environmental effects of ammonium on the methanotrophic activity. Download Text S1, DOCX file, 0.03 MB. Copyright © 2022 Guo et al. 2022 Guo et al. https://creativecommons.org/licenses/by/4.0/ This content is distributed under the terms of the Creative Commons Attribution 4.0 International license.

The inhibitory effects of NH_3_ oxidation by pMMO on methanotrophic activity occur through toxic nitrogen products such as NH_2_OH and nitrite (NO_2_^−^). Although the affinity of pMMO for NH_3_ is generally lower than that for CH_4_ (20), aerobic methanotrophs with high tolerance to these nitrogen products need the ability to quickly detoxify them by both nitrifying and denitrifying processes (19, 26). Both the detailed survey of genes involved in nitrogen metabolism in methanotrophic bacteria and physiological studies suggest that methanotrophs with efficient hydroxylamine detoxification pathways show increased competitiveness under high NH_4_^+^-N conditions (27–29). Nevertheless, the acclimatization response of methanotrophs to an increasing NH_4_^+^ load has not yet been conclusively understood at the cellular level. This is particularly valid for type IIa methanotrophs and more specifically for *Methylocystis* spp.

**FIG 1:**
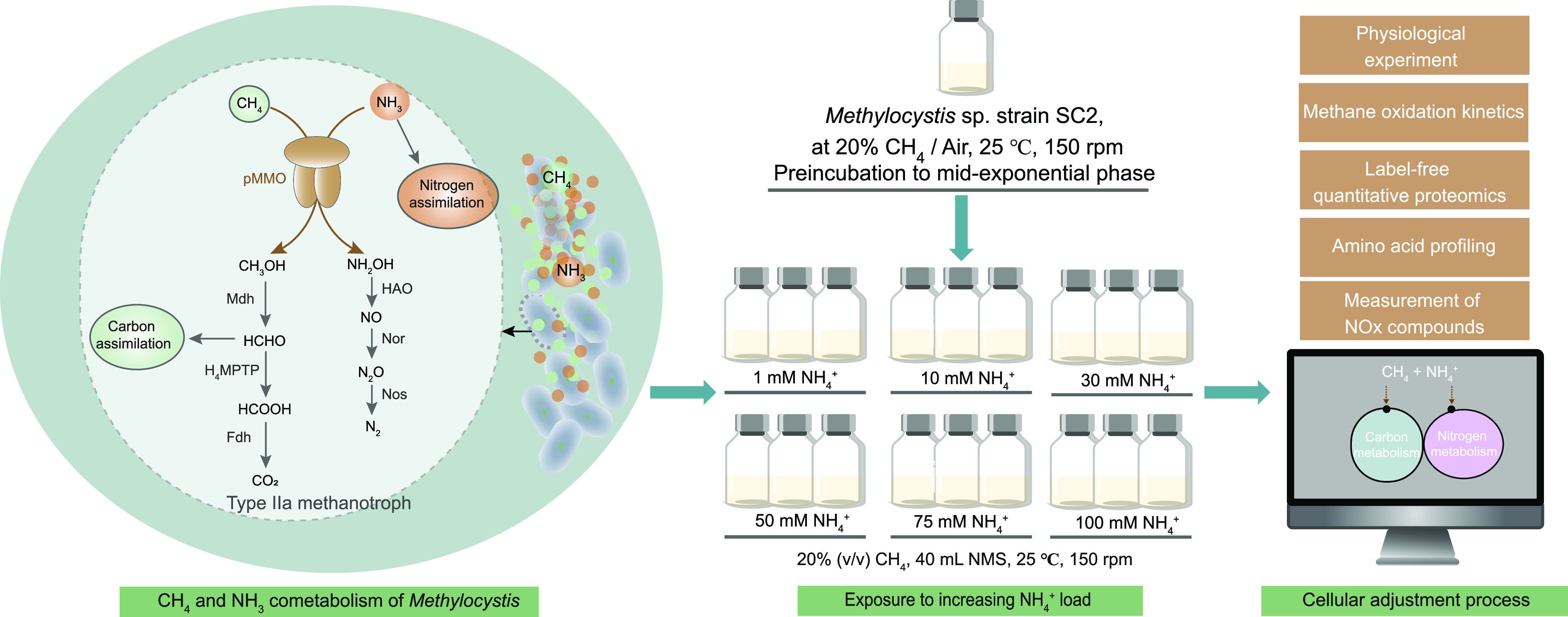
Experimental design setup to elucidate the physiological and cellular responses of *Methylocystis* sp. strain SC2 to an increasing NH_4_^+^ load. Detailed information on the experimental approach is given in Materials and Methods.

Therefore, we here aimed to elucidate the cellular mechanisms underlying the acclimatization response of *Methylocystis* sp. strain SC2 to an increasing NH_4_^+^ load. In particular, we aimed (i) to determine the NH_4_^+^ threshold level to which strain SC2 is able to acclimatize and (ii) to assess the cellular adjustment processes triggered by this threshold level. We expected to observe a dual response of strain SC2, with the first one being a general response to increasing ionic-osmotic stress and the second one being a methanotroph-specific response to hydroxylamine stress. Recently, we developed a new analytical proteomics workflow for strain SC2 which captures 62% of the predicted SC2 proteome under standard growth conditions (30). This workflow tackles the major challenges related to the large amount of integral membrane proteins that need to be efficiently solubilized and digested for downstream analysis. Thus, our research combined SC2 growth experiments under an increasing NH_4_^+^ load (1 to 100 mM) with global proteomics, analysis of intracellular amino acids (metabolomics), and measurement of nitrogen oxides compounds (Fig. 1).

## RESULTS

The following subsections describe the effects of an increasing NH_4_^+^ load on the activity of *Methylocystis* sp. strain SC2. This involves the impact on its growth response and apparent *K_m_* value of CH_4_ oxidation, the global proteome, and the concentration of intracellular amino acids. The effects of 10, 30, 50, and 75 mM NH_4_^+^ on the physiology and global proteome of strain SC2 were inferred by comparison to the reference standard growth conditions (1 mM NH_4_^+^). Finally, we quantified NO_2_^−^ and nitrous oxide (N_2_O) production by strain SC2 in relation to both an increasing NH_4_^+^ load and incubation time.

### Growth response.

**FIG 2:**
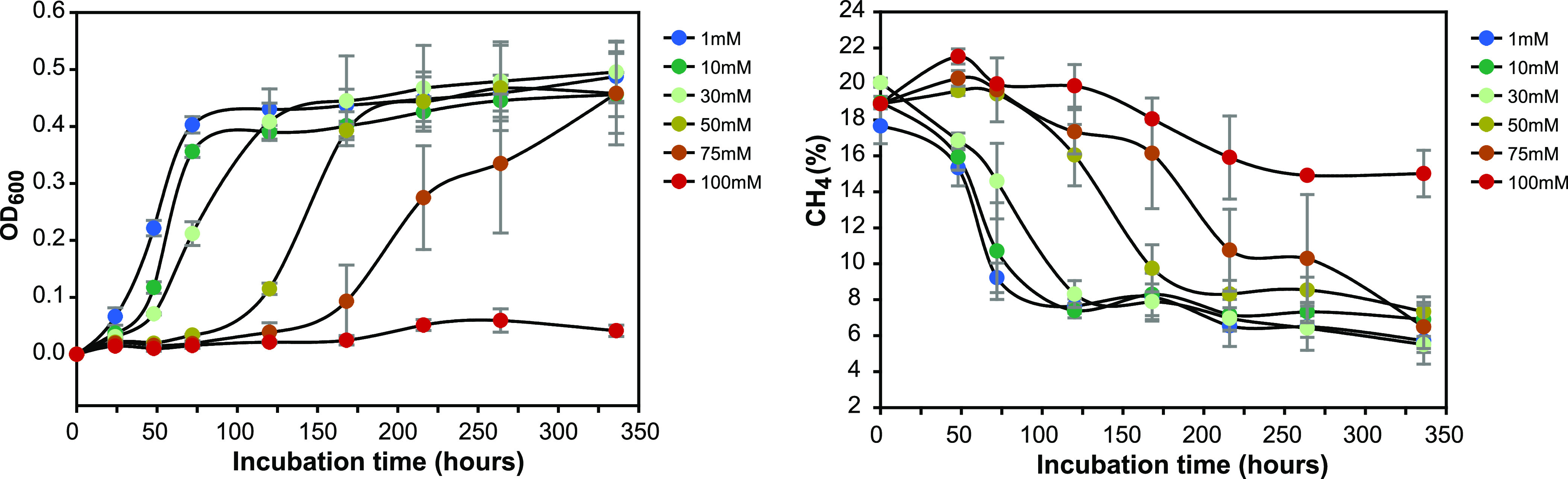
Effect of increasing NH_4_^+^ concentrations on growth of (left) and CH_4_ consumption by (right) *Methylocystis* sp. strain SC2. Growth (OD_600_) and CH_4_ concentration were regularly monitored over the whole incubation period. Measurements were done in triplicate cultures. Growth response occurred with up to 75 mM NH_4_Cl, corresponding to a total ionic medium strength of 114 mM (see Table S2 in the supplemental material). No growth occurred with 100 mM NH_4_Cl, corresponding to a total ionic medium strength of 139 mM. Error bars show standard deviations of the results of triplicate cultures.

To assess increasing concentration levels of ammonium on SC2 growth, cells of strain SC2 were incubated in strict batch incubation mode with a CH_4_-air mixing ratio of 20:80 (vol/vol) (Fig. 2). Cell density (optical density at 600 nm [OD_600_]) and the headspace concentrations of both CH_4_ and CO_2_ were regularly measured during the complete incubation period of up 336 h (14 days), ranging from early lag phase to late stationary phase (Fig. 2). The addition of 1 mM NH_4_^+^ prompted immediate growth of strain SC2, while the addition of 10 mM and 30 mM NH_4_^+^ also had nearly no delay effect on the growth response of strain SC2 (Fig. 2). Supplementation with NH_4_^+^ levels higher than 30 mM triggered significant delays in the growth response, with lag phase durations of 75 h (50 mM NH_4_^+^) and 125 h (75 mM NH_4_^+^). This was linked to a significant decrease in the growth and CH_4_ consumption rates (Fig. 2 and Table 1). However, regardless of the amount of ammonium added (1 to 75 mM NH_4_^+^), all SC2 cultures grew to the same final OD_600_ of about 0.45 (Fig. 2). Accordingly, the total cell dry weight (CDW), the total amount of CH_4_ consumed, and, in consequence, the biomass yield did not significantly differ between the different NH_4_^+^ concentrations. However, the standard deviation of CDW increased with increasing NH_4_^+^ load, thereby suggesting an increasingly heterogeneous population response (Table 1). There was no significant CH_4_ consumption or cell density change after the addition of 100 mM NH_4_^+^ (Fig. 2).

**TABLE 1:** Physiological growth parameters of *Methylocystis* sp. strain SC2 during cultivation under different NH_4_^+^ concentrations^*a*^

Ammonium treatment	CDW^*b*^ (mg)	Growth rate (mg CDW/day)	CH_4_ consumption (mmol CH_4_)	Biomass yield (mg CDW/mmol CH_4_)	CH_4_ consumption rate (mmol CH_4_/g CDW/day)
1 mM	3.5 ± 0.04	1.75 ± 0.02	0.29 ± 0.04	12.38 ± 1.59	40.87 ± 5.55
10 mM	3.32 ± 0.13	1.66 ± 0.07	0.27 ± 0.03	12.29 ± 1.72	41.22 ± 5.68
30 mM	3.93 ± 0.35	1.31 ± 0.12**	0.28 ± 0.01	14.08 ± 1.04	23.75 ± 1.74**
50 mM	4.26 ± 0.54	0.71 ± 0.09***	0.36 ± 0.02	11.84 ± 2.27	14.42 ± 2.68***
75 mM	4.36 ± 1.02	0.48 ± 0.11***	0.35 ± 0.04	12.4 ± 3.21	9.34 ± 2.2***

a All growth parameters were calculated based on triplicate cultures. Asterisks indicate significant differences at *P* values of ≤0.05 (*),  ≤0.01 (**), and ≤0.001 (***) relative to the control treatment (1 mM NH_4_^+^), using Tukey's method with one-way ANOVA.

b Cell dry weight (CDW) was calculated using 1 OD_600_ = 0.26 g CDW/L of strain SC2 culture (68).

10.1128/msystems.00403-22.4 TABLE S2 Growth medium: ionic strength, pH, dissolved CH_4_, aqueous NH_3_, and CH_4_/NH_3_ ratio across the NH_4_Cl treatments. Download Table S2, PDF file, 0.08 MB. Copyright © 2022 Guo et al. 2022 Guo et al. https://creativecommons.org/licenses/by/4.0/ This content is distributed under the terms of the Creative Commons Attribution 4.0 International license.

### Apparent *K_m_* value of CH_4_ oxidation.

The CH_4_-air mixing ratios adjusted to 10:90 and 20:80 (vol/vol) showed no significant difference in SC2 growth response when supplemented with 10 mM, 30 mM, and 50 mM NH_4_^+^. No increase in cell density (OD_600_) was observed in SC2 cultures supplemented with 50 mM NH_4_^+^ after the CH_4_-air mixing ratio was adjusted to below 10:90 (vol/vol) (see Fig. S1 at https://doi.org/10.6084/m9.figshare.20750236.v3).

The SC2 growth parameters determined for the different CH_4_-air mixing ratios and increasing NH_4_^+^ concentrations revealed that relative to the control (20:80 [vol/vol]), ratio values of 5:95 and 2.5:97.5 (vol/vol) reduced the CDW production, growth rate, CH_4_ consumption, and CH_4_ consumption rate. This was significant (*P < *0.001) across all physiological growth parameters for SC2 cultures supplemented with 50 mM NH_4_^+^ (see Table S1 in the supplemental material). The increase in NH_4_^+^ concentration greatly altered the apparent *K_m_* values [*K_m_*_(app)_] for CH_4_ oxidation, being 0.17 μM, 1.20 μM, and 1.40 μM under growth conditions with 10 mM, 30 mM, and 50 mM NH_4_^+^, respectively (Table S2). This corresponds well to the decrease in the ratio of CH_4_ to NH_3_ dissolved in the liquid growth medium (Table S2). The *K_m_*_(app)_ value for 75 mM NH_4_^+^ could not be calculated, because growth of strain SC2 was completely inhibited when incubated under a headspace of 2.5% and 5% CH_4._

10.1128/msystems.00403-22.3 TABLE S1 Physiological parameter of *Methylocystis* sp. strain SC2 measured in response to various CH_4_-air mixing ratios under increasing NH_4_^+^ concentrations (see Fig. S1 at https://doi.org/10.6084/m9.figshare.20750236.v3 for a graphic presentation of the physiological growth parameters). Download Table S1, PDF file, 0.1 MB. Copyright © 2022 Guo et al. 2022 Guo et al. https://creativecommons.org/licenses/by/4.0/ This content is distributed under the terms of the Creative Commons Attribution 4.0 International license.

### Whole-cell proteome.

**FIG 3:**
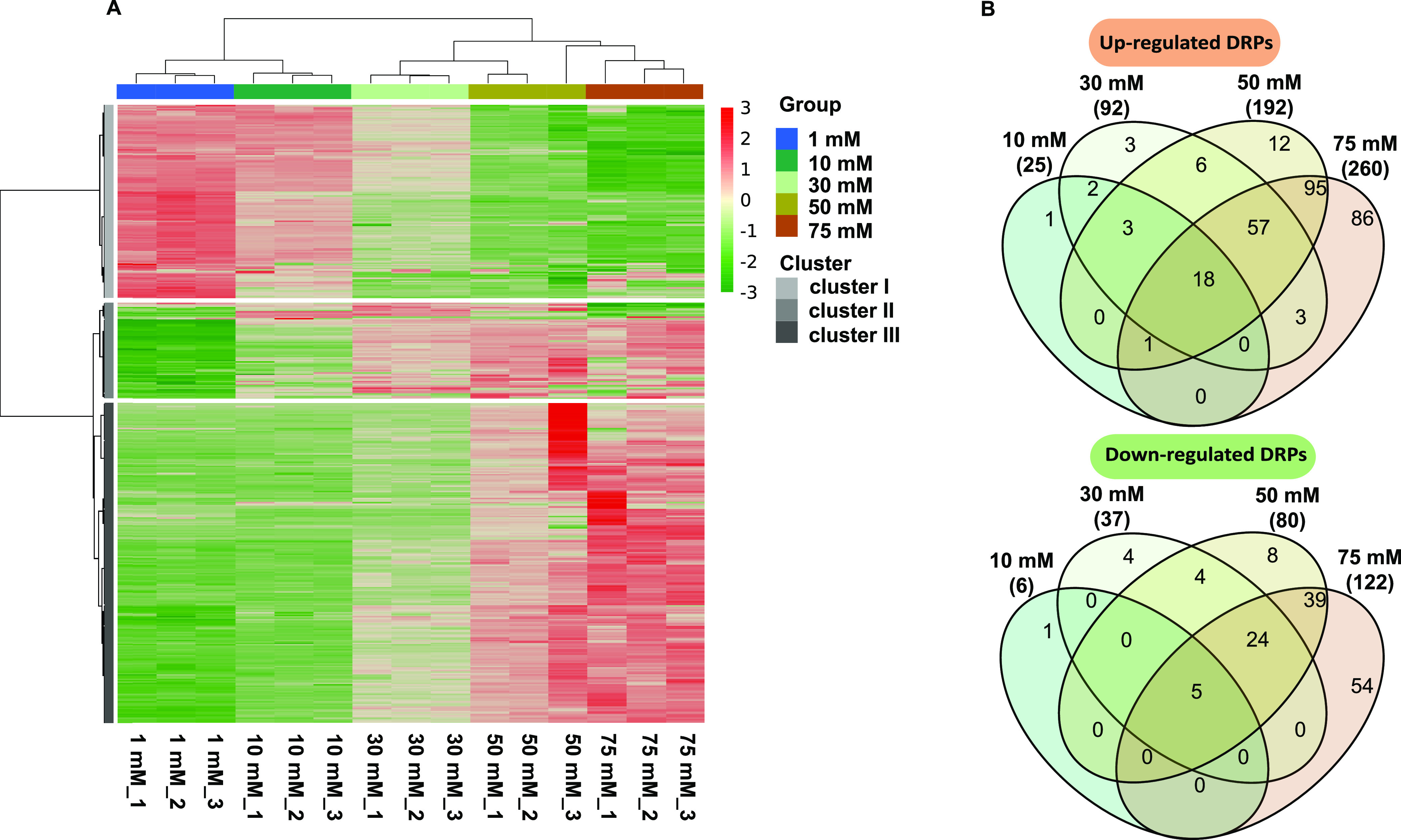
Comparative analysis of the global LFQ proteomes. (A) Heat map showing the DRP pattern of each replicate culture in response to increasing NH_4_^+^ concentrations (1 mM, 10 mM, 30 mM, 50 mM, and 75 mM NH_4_^+^). Using Euclidean distances, the heat map was built based on the LFQ intensities of 438 DRPs. The color scale indicates Z-score-normalized LFQ intensity values. (B) Venn diagram showing the overlap of up- and downregulated DRPs among the five different NH_4_^+^ treatments. Further details can be found in Data Set S1 in the supplemental material.

Global proteomics led to the detection of 2,206 proteins, of which 438 proteins were identified to be differentially regulated proteins (DRPs) in at least one of the NH_4_^+^ treatments (Data Set S1). The 438 DRPs cover 10.8% of the total SC2 proteome (4,040 proteins) deposited in the UniProt database (https://www.uniprot.org/taxonomy/187303). Neither the PmoCAB1 subunits of low-affinity pMMO1 nor the PmoB2 subunit of the high-affinity pMMO2 showed a differential regulation. The PmoC2 and PmoA2 subunits of pMMO2 were not detectable at any of the NH_4_^+^ treatment concentrations (Table 2). Hierarchical cluster analysis and Pearson correlation coefficient values showed highly reproducible DRP profiles for all five NH_4_^+^ conditions (Fig. 3A; see also Fig. S2 at https://doi.org/10.6084/m9.figshare.20750236.v3). The heat map of sample-to-sample distances showed high similarities between the DRP profiles of the 1 mM and 10 mM NH_4_^+^ treatments, but in particular between those of the 50 mM and 75 mM NH_4_^+^ treatments. More specifically, the DRPs grouped into three distinct clusters comprising a total of 141, 65, and 232 proteins, respectively. The 232 proteins of DRP cluster III were significantly upregulated only under 50 mM and 75 mM NH_4_^+^ conditions (Fig. 3A; see also Fig. S3 at https://doi.org/10.6084/m9.figshare.20750236.v3 and Data Set S1 in the supplemental material). High DRP profile similarities between the 1 mM/10 mM and 50 mM/75 mM NH_4_^+^ comparisons were further evidenced by the results of principal-component analysis (PCA) (see Fig. S4 at https://doi.org/10.6084/m9.figshare.20750236.v3).

**TABLE 2:** Differentially regulated proteins involved in the PPI network^*a*^

Function category	UniProt ID	Gene	Protein description	Median no. of DRPs at NH_4_^+^ treatment of:					Log_2_ ratio value^*b*^ at NH_4_^+^ treatment of:				*q* value^*b*^ at NH_4_^+^ treatment of:			
				1 mM	10 mM	30 mM	50 mM	75 mM	10 mM	30 mM	50 mM	75 mM	10 mM	30 mM	50 mM	75 mM
Methane metabolism																
Methane oxidation	J7QM98	*pmoC1*	Particulate methane monooxygenase (PmoC1)	1.83E + 08	2.04E + 08	2.30E + 08	2.62E + 08	2.61E + 08	0.163	0.334	0.519	0.515	0.072	0.001	0.001	0.001
	Q70EF3	*pmoA1*	Particulate methane monooxygenase (PmoA1)	3.10E + 08	3.51E + 08	3.85E + 08	4.26E + 08	4.44E + 08	0.178	0.311	0.456	0.516	0.036	0.001	0.001	0.003
	Q70EF2	*pmoB1*	Particulate methane monooxygenase (PmoB1)	1.41E + 10	1.63E + 10	1.98E + 10	2.09E + 10	2.11E + 10	0.206	0.488	0.566	0.583	0.012	0.000	0.000	0.005
	Q6MZ16	*pmoB2* ^*c*^	Particulate methane monooxygenase (PmoB2)	2.80E + 06	2.71E + 06	3.63E + 06	2.76E + 06	1.72E + 06	−0.046	0.375	−0.024	−0.707	0.980	0.000	0.366	0.000
	J7QLZ6	*pmoC2_Gs_*	Chromosome-encoded PmoC2_Gs_	4.50E + 05	8.10E + 05	9.95E + 05	1.67E + 06	2.23E + 06	0.848	**1.145**	**1.894**	**2.309**	0.036	0.005	0.001	0.000
	I4EB56 ^*d*^	*pmoC3_Ps_*	Plasmid-borne PmoC3_Ps_	5.76E + 07	5.60E + 07	2.07E + 07	2.07E + 06	2.57E + 06	−0.040	−1.477	−4.800	−4.487	0.601	0.000	0.000	0.000
Methanol metabolism	J7Q447	*xoxF*	PQQ-dependent dehydrogenase, methanol/ethanol family	1.97E + 08	1.43E + 08	7.08E + 07	9.45E + 07	1.48E + 08	−0.463	−1.473	−1.057	−0.409	0.001	0.000	0.003	0.064
	J7QNA0	*xoxG*	Putative cytochrome *c* protein	1.01E + 07	8.33E + 06	6.54E + 06	5.32E + 06	4.12E + 06	−0.276	−0.624	−0.923	−1.290	0.182	0.001	0.002	0.002
	J7Q898	*xoxJ*	Extracellular solute-binding protein family 3	9.69E + 05	6.89E + 05	4.60E + 05	5.17E + 05	8.72E + 05	−0.492	−1.075	−0.907	−0.153	0.006	0.000	0.003	0.401
	J7QHX8	*mxaF*	Methanol dehydrogenase MxaF	5.64E + 09	6.84E + 09	9.08E + 09	1.04E + 10	1.14E + 10	0.279	0.688	0.879	**1.022**	0.003	0.000	0.000	0.000
	J7Q9K7	*mxaJ*	Extracellular solute-binding protein family 3	2.87E + 08	4.54E + 08	6.79E + 08	6.45E + 08	7.15E + 08	0.662	**1.242**	**1.170**	**1.318**	0.000	0.000	0.001	0.000
	J7QR86	*mxaG*	Cytochrome *c* class I	2.64E + 08	2.81E + 08	2.75E + 08	2.33E + 08	2.25E + 08	0.089	0.056	−0.183	−0.235	0.053	0.264	0.009	0.104
	J7Q4V7	*mxaI*	Methanol dehydrogenase (cytochrome *c*) subunit 2	2.99E + 08	3.54E + 08	5.11E + 08	5.75E + 08	5.28E + 08	0.246	0.774	0.945	0.820	0.087	0.001	0.000	0.001
	J7QUZ4	*mxaR*	ATPase associated with various cellular activities AAA_3	3.76E + 07	3.95E + 07	4.51E + 07	5.05E + 07	5.83E + 07	0.071	0.263	0.427	0.632	0.328	0.025	0.001	0.000
	J7QHY1	*mxaS*	Uncharacterized protein	8.32E + 05	7.81E + 05	9.26E + 05	1.19E + 06	1.41E + 06	−0.092	0.155	0.522	0.766	0.279	0.199	0.001	0.000
	J7Q9K9	*mxaA*	MxaA protein, putative	6.81E + 06	7.70E + 06	9.41E + 06	1.09E + 07	1.30E + 07	0.177	0.465	0.681	0.936	0.022	0.000	0.000	0.000
	J7QR90	*mxaC*	von Willebrand factor type A	9.12E + 05	1.09E + 06	1.37E + 06	1.55E + 06	1.87E + 06	0.261	0.586	0.764	**1.034**	0.003	0.000	0.000	0.000
	J7Q4V8	*mxaK*	Uncharacterized protein	2.59E + 06	2.91E + 06	3.71E + 06	4.60E + 06	5.62E + 06	0.167	0.519	0.829	**1.117**	0.799	0.019	0.003	0.000
	J7QUZ9	*mxaL*	von Willebrand factor type A	9.13E + 05	1.04E + 06	1.36E + 06	1.59E + 06	2.07E + 06	0.186	0.574	0.803	**1.183**	0.016	0.000	0.000	0.000
	J7QHY3	*mxaD*	MxaD protein/polyketide cyclase/dehydrase	2.08E + 08	2.44E + 08	3.28E + 08	4.14E + 08	4.72E + 08	0.235	0.660	0.995	**1.186**	0.008	0.000	0.000	0.000
	J7Q9L1	*mxaH*	Uncharacterized protein	1.20E + 06	1.49E + 06	2.17E + 06	2.69E + 06	3.41E + 06	0.315	0.856	**1.168**	**1.511**	0.010	0.000	0.000	0.000
Stress response																
Potassium transport	J7QR19	*kdpC*	Potassium-transporting ATPase KdpC subunit	4.98E + 06	4.25E + 06	5.24E + 06	7.86E + 06	1.49E + 07	−0.229	0.073	0.658	**1.583**	0.010	0.102	0.000	0.000
	J7Q4U0	*kdpB*	Potassium-transporting ATPase ATP-binding subunit	4.19E + 06	4.26E + 06	5.25E + 06	8.35E + 06	1.61E + 07	0.023	0.326	0.994	**1.946**	0.998	0.000	0.000	0.000
	J7QUT1	*kdpA*	Potassium-transporting ATPase potassium-binding subunit	1.21E + 06	1.07E + 06	1.27E + 06	1.78E + 06	3.63E + 06	−0.176	0.064	0.548	**1.579**	0.019	0.470	0.002	0.000
	J7Q9H9	*BN69_2486*	Osmosensitive K channel His kinase sensor	1.12E + 05	1.46E + 05	1.80E + 05	3.19E + 05	5.11E + 05	0.393	0.691	**1.514**	**2.196**	0.061	0.005	0.000	0.000
General stress-induced proteins	J7QQ02	*BN69_2097*	Stress response DNA-binding protein (Dps)	9.02E + 06	8.97E + 06	1.39E + 07	5.04E + 07	6.11E + 07	−0.008	0.628	**2.483**	**2.759**	0.366	0.000	0.002	0.000
	J7QF27	*BN69_0580*	Heat shock protein Hsp20	5.17E + 07	3.09E + 07	6.47E + 07	1.63E + 08	2.52E + 08	−0.744	0.323	**1.656**	**2.283**	0.000	0.002	0.001	0.000
	J7QJH1	*BN69_3580*	Heat shock protein Hsp20	1.03E + 07	8.09E + 06	6.06E + 06	5.01E + 06	6.47E + 06	−0.344	−0.762	−1.035	−0.667	0.002	0.000	0.000	0.000
	J7QM64	*htpG*	Chaperone protein HtpG	1.92E + 08	1.50E + 08	1.14E + 08	9.54E + 07	9.36E + 07	−0.356	−0.744	−1.006	−1.033	0.001	0.000	0.000	0.000
	J7QPM2	*hdeA*	Probable acid stress chaperone HdeA	2.42E + 07	3.26E + 07	4.32E + 07	5.79E + 07	6.73E + 07	0.428	0.834	**1.257**	**1.474**	0.007	0.000	0.000	0.000
	J7Q5H9	*BN69_3658*	Alcohol dehydrogenase GroES domain protein	1.20E + 04	1.17E + 04	1.72E + 04	9.07E + 04	4.12E + 04	−0.035	0.521	**2.920**	**1.783**	0.883	0.651	0.028	0.114
	J7QV15	*BN69_2599*	CsbD family protein	9.87E + 05	1.19E + 06	1.43E + 06	3.12E + 06	1.00E + 07	0.274	0.531	**1.659**	**3.342**	0.183	0.009	0.010	0.000
	J7QPM2	*hdeA*	Probable acid stress chaperone HdeA	2.42E + 07	3.26E + 07	4.32E + 07	5.79E + 07	6.73E + 07	0.428	0.834	**1.257**	**1.474**	0.007	0.000	0.000	0.000
	J7QHI8	*BN69_2205*	Stress-induced protein	2.74E + 05	3.18E + 05	3.44E + 05	2.20E + 06	1.71E + 06	0.212	0.327	**3.000**	**2.637**	0.189	0.024	0.001	0.000
Glutathione metabolism	J7QSL3	*BN69_1574*	Glutathione peroxidase	2.47E + 06	2.67E + 06	3.86E + 06	5.68E + 06	7.39E + 06	0.115	0.646	**1.202**	**1.582**	0.315	0.000	0.000	0.000
	J7QTI3	*BN69_1939*	Glutathione *S*-transferase, N-terminal domain	1.44E + 05	1.73E + 05	2.37E + 05	5.12E + 05	6.53E + 05	0.260	0.716	**1.825**	**2.176**	0.082	0.010	0.002	0.000
	J7QHL1	*BN69_2280*	Glutathione *S*-transferase domain protein	1.82E + 05	2.21E + 05	2.05E + 05	2.69E + 05	4.66E + 05	0.283	0.178	0.569	**1.361**	0.147	0.016	0.163	0.000
	J7Q532	*BN69_2948*	Glutathione *S*-transferase domain protein	8.02E + 04	1.13E + 05	1.62E + 05	1.78E + 05	1.44E + 05	0.495	**1.013**	**1.151**	0.842	0.045	0.003	0.002	0.003
	J7QIW6	*BN69_3230*	Glutathione *S*-transferase domain protein	3.56E + 07	2.48E + 07	1.67E + 07	1.26E + 07	1.02E + 07	−0.522	−1.088	−1.501	−1.800	0.000	0.000	0.000	0.000
	J7QA29	*BN69_3006*	ChaC family protein	5.05E + 05	6.39E + 05	8.44E + 05	9.33E + 05	1.15E + 06	0.339	0.740	0.886	**1.190**	0.007	0.000	0.000	0.000
	J7QQ61	*BN69_0634*	Glutamate/cysteine ligase	4.98E + 07	4.86E + 07	3.86E + 07	2.96E + 07	2.30E + 07	−0.035	−0.366	−0.749	−1.117	0.495	0.000	0.000	0.000
Synthesis of amino acids/compatible solutes	J7QVE2	*argD*	Acetylornithine aminotransferase	7.62E + 04	8.88E + 04	1.17E + 05	1.81E + 05	2.11E + 05	0.220	0.623	**1.251**	**1.469**	0.775	0.009	0.002	0.000
	J7QHB7	*BN69_2040*	Glycine dehydrogenase (aminomethyl transferring)	9.20E + 05	6.17E + 05	6.61E + 05	1.52E + 06	2.50E + 06	−0.577	−0.478	0.725	**1.440**	0.005	0.003	0.005	0.002
	J7QPV5	*BN69_2037*	Aminomethyltransferase	4.52E + 05	3.38E + 05	3.23E + 05	7.33E + 05	1.12E + 06	−0.418	−0.484	0.699	**1.311**	0.003	0.003	0.002	0.001
	J7Q4N1	*BN69_2223*	5-Aminolevulinate synthase	2.82E + 06	3.34E + 06	7.16E + 06	8.20E + 06	9.09E + 06	0.241	**1.341**	**1.537**	**1.685**	0.079	0.000	0.053	0.036
Nitrogen metabolism																
Ammonium transport and assimilation	J7QLT5	*BN69_0652*	Glutamine synthetase	1.65E + 09	1.61E + 09	1.61E + 09	1.56E + 09	1.57E + 09	−0.039	−0.034	−0.082	−0.077	0.212	0.266	0.100	0.343
	J7QQX7	*BN69_0914*	Nitrogen regulatory protein P-II	4.89E + 06	3.55E + 06	2.47E + 06	1.99E + 06	2.38E + 06	−0.464	−0.987	−1.295	−1.042	0.027	0.000	0.000	0.000
	J7QFK3	*BN69_0915*	Ammonium transporter	1.06E + 06	5.86E + 05	6.69E + 05	4.75E + 05	6.70E + 05	−0.857	−0.664	−1.159	−0.663	0.050	0.010	0.002	0.015
	J7QFL3	*BN69_0930*	Nitrogen regulatory protein P-II	2.11E + 06	9.85E + 05	8.62E + 05	7.17E + 05	9.07E + 05	−1.101	−1.292	−1.559	−1.220	0.000	0.000	0.000	0.000
	J7QR56	*BN69_0999*	Glutamate dehydrogenase	4.30E + 04	2.97E + 04	1.14E + 05	2.78E + 05	4.04E + 05	−0.534	**1.406**	**2.693**	**3.231**	0.689	0.001	0.002	0.000
	J7QTS4	*BN69_3582*	Glutamate synthase [NADH], amyloplastic	1.71E + 08	1.79E + 08	1.80E + 08	1.83E + 08	1.93E + 08	0.065	0.077	0.096	0.173	0.323	0.141	0.064	0.018
	J7QX35	*BN69_3584*	Glutamate synthase, NADH/NADPH, small subunit	7.65E + 07	7.81E + 07	7.73E + 07	7.78E + 07	8.57E + 07	0.030	0.014	0.025	0.163	0.942	0.908	0.944	0.054
Hydroxylamine detoxification	J7Q787	*hcp*	Hybrid cluster protein	3.29E + 08	8.67E + 08	1.05E + 09	1.12E + 09	1.08E + 09	**1.399**	**1.674**	**1.763**	**1.722**	0.000	0.000	0.000	0.000
	I4EBE8 ^*d*^	*hcp*	Hybrid cluster protein	1.93E + 05	5.50E + 05	6.87E + 05	7.27E + 05	5.60E + 05	**1.515**	**1.835**	**1.917**	**1.538**	0.001	0.000	0.000	0.001
	J7QAB0	*haoB*	Putative HaoB	1.78E + 07	3.78E + 07	4.71E + 07	5.09E + 07	5.55E + 07	**1.086**	**1.404**	**1.516**	**1.638**	0.000	0.000	0.000	0.000
	J7QSX9	*haoA*	Hydroxylamine oxidase, HaoA	5.62E + 06	1.76E + 07	2.40E + 07	3.01E + 07	3.37E + 07	**1.646**	**2.095**	**2.420**	**2.584**	0.000	0.000	0.000	0.000

a The complete list of the 121 PPI network proteins is shown in Data Set S4 at https://doi.org/10.6084/m9.figshare.20556651.v1. In this table, significantly upregulated proteins are shown in bold, while significantly downregulated proteins are underlined.

b Both log_2_ ratio values and *q* values are given relative to the 1 mM treatment.

c Note that the LFQ values of PmoB2 were 3 to 4 orders of magnitude lower than those of PmoB1. The LFQ values of PmoC2 and PmoA2 were below the detection limit across all NH_4_^+^ treatments. The fact that LFQ values of PmoC and PmoA subunits were lower than those of PmoB, as observed for both pMMO isozymes (pMMO1 and pMMO2), may be due the fact that PmoA and PmoC are integral membrane subunits, thereby resulting in low solubilization efficiency. In contrast, PmoB comprises two periplasmic domains connected by two transmembrane helices (75, 76). A significant downregulation of *pmoCAB2* at the transcriptome level was observed after transfer of a batch of mid-log-phase SC2 cells pregrown on 10 mM NMS (nitrate-based mineral medium) to the same medium containing 15 mM (NH_4_)_2_SO_4_ (30 mM NH_4_^+^) instead of 10 mM NO_3_-. The SC2 cells were incubated for 10 h on 15 mM (NH_4_)_2_SO_4_ prior to sampling for RNA extraction (34).

d Proteins encoded by plasmid-borne genes.

10.1128/msystems.00403-22.5 DATA SET S1 Complete list of all 438 proteins that were differentially regulated in at least one NH_4_^+^ treatment. Download Data Set S1, XLSX file, 0.1 MB. Copyright © 2022 Guo et al. 2022 Guo et al. https://creativecommons.org/licenses/by/4.0/ This content is distributed under the terms of the Creative Commons Attribution 4.0 International license.

A certain number of DRPs were coregulated regardless of the initial NH_4_^+^ concentration. A total of 18 DRPs (10 mM NH_4_^+^), 57 DRPs (30 mM NH_4_^+^), and 95 DRPs (50 mM NH_4_^+^) were co-upregulated under the 75 mM NH_4_^+^ condition (Fig. 3B; Data Set S1). A similar pattern was observed for the downregulated DRPs, with 5 DRPs (10 mM NH_4_^+^), 24 DRPs (30 mM NH_4_^+^), and 39 DRPs (50 mM NH_4_^+^) being co-downregulated under the 75 mM NH_4_^+^ condition (Fig. 3B; Data Set 1). Totals of 86 and 54 DRPs were found to be significantly up- and downregulated, respectively, only under the 75 mM NH_4_^+^ condition. This corresponds to more than 30% (140/438) of the total identified DRPs (Fig. 3B; Data Set S1). The number of DRPs showed a significant and positive relationship with the increase in NH_4_^+^ load for both up- and downregulated proteins (*r*^2^ values of 0.99) (see Fig. S5 at https://doi.org/10.6084/m9.figshare.20750236.v3 and Data Set S2 at https://doi.org/10.6084/m9.figshare.20556600.v1).

### Functional categorization of differentially regulated proteins.

**FIG 4:**
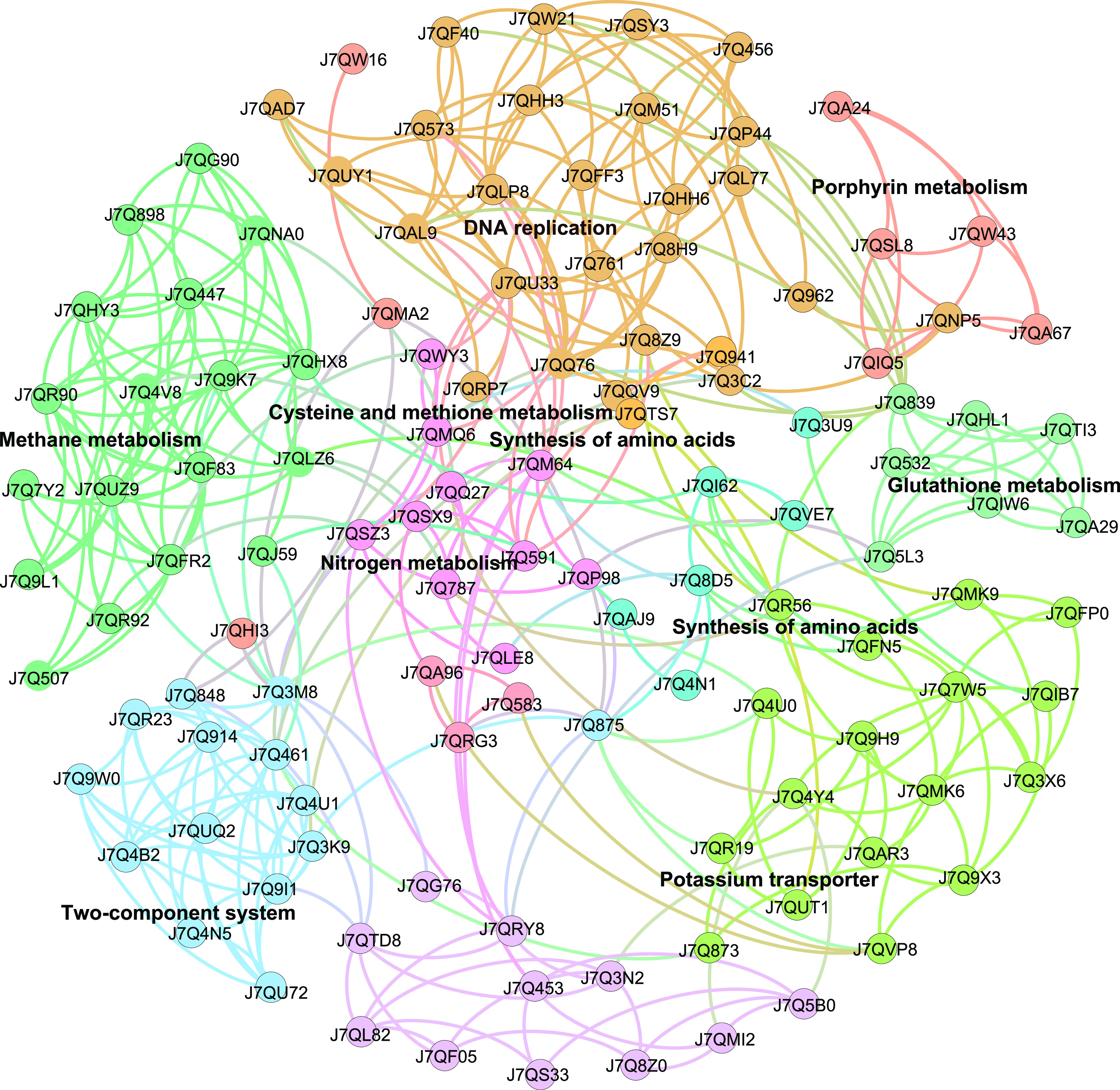
PPI network of 121 DRPs that are connected by a total of 431 edges. The proteins were partitioned into 10 highly connected functional modules, which are highlighted by different colors using the modularity class methods in Gephi. The functional categorization of the modules is based on KEGG level 3, except for general stress response and potassium transport. The size of nodes and edges is proportional to the number of connections (its degree). The protein identity of each node is indicated by the UniProt ID. The network edges indicate both functional and physical protein associations based on active interaction sources, including text mining, experiments, databases, coexpression, neighborhood, gene fusion, and cooccurrence. See Data Set S4 at https://doi.org/10.6084/m9.figshare.20556651.v1 for details on the 121 DRPs used to construct the PPI network.

Among the 438 DRPs, functional information was available for 312 DRPs by their UniProt identifiers (Data Set S1). The remaining 126 DRPs were uncharacterized proteins based on UniProt. A survey of the 312 functionally predicted DRPs against the Kyoto Encyclopedia of Genes and Genomes (KEGG) database allowed us to annotate a total of 95 DRPs (see Fig. S6 at https://doi.org/10.6084/m9.figshare.20750236.v3 and Data Set S3 at https://doi.org/10.6084/m9.figshare.20556633.v1). A protein-protein interaction (PPI) network analysis revealed 121 proteins to be highly interactive (Fig. 4). These were partitioned into 10 functional modules, including methane metabolism, nitrogen metabolism, stress response proteins, potassium transport, biosynthesis of amino acids, glutathione metabolism, transporters, porphyrin (cytochrome) metabolism, and DNA replication (Fig. 4). A selection of 56 DPRs is shown in Table 2, while information on the complete set of 121 proteins can be found in Data Set S4 at https://doi.org/10.6084/m9.figshare.20556651.v1. In addition to proteins related to glutathione metabolism and DNA replication, those involved in nitrogen metabolism were particularly enriched at high NH_4_^+^ concentrations (50 mM NH_4_^+^, *q* value < 0.05; and 75 mM NH_4_^+^, *P* value < 0.05) (see Fig. S7 at https://doi.org/10.6084/m9.figshare.20750236.v3). Nitrogen metabolism included proteins involved in NH_4_^+^ transport and assimilation and in hydroxylamine detoxification (Table 2).

### Amino acid profiling.

**FIG 5:**
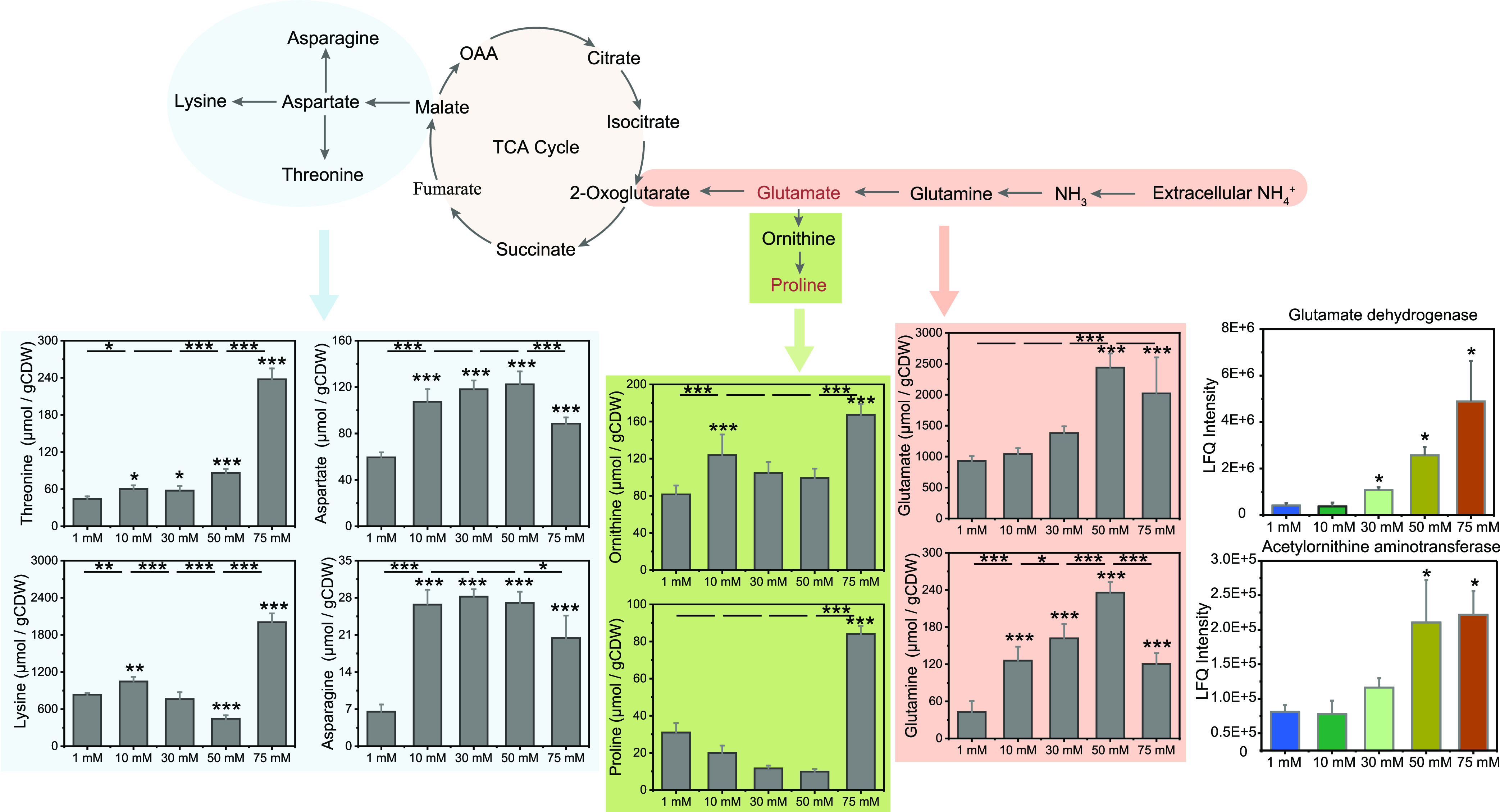
Amino acids that show a statistically significant change in their intracellular concentrations in response to increasing NH_4_^+^ levels. The association between particular pathway information (upper panel) and the results of amino acid profiling (lower panel) is indicated by an arrow and the same background color. Error bars indicate standard deviations of results of replicate cultures (*n* = 4). The lower-panel asterisks indicate significant differences (*P* value ≤ 0.05) relative to the control treatment (1 mM NH_4_^+^). The upper-panel asterisks indicate significant differences between the stepwise increase in NH_4_^+^ load. Significant difference was calculated using the one-way analysis of variance (ANOVA) Holm-Sidak method: *, *P* value ≤ 0.05; **, *P* value ≤ 0.01; ***, *P* value ≤ 0.001.

Of the 16 amino acids detected, 15 amino acids showed a significant change in their intracellular concentrations across the five NH_4_^+^ treatments (see Data Set S5 at https://doi.org/10.6084/m9.figshare.20750203.v2). In particular, the intracellular concentration of glutamate significantly increased to 2,438.69 μmol/g CDW under the 50 mM NH_4_^+^ condition but slightly decreased to 2,020.59 μmol/g CDW under the 75 mM NH_4_^+^ condition (Fig. 5; see also Data Set S5 at https://doi.org/10.6084/m9.figshare.20750203.v2). Glutamine also showed the greatest intracellular accumulation at 50 mM NH_4_^+^, with 235.69 μmol/g CDW. Unlike with glutamate and glutamine, the intracellular concentration of proline significantly decreased from 1 mM to 50 mM NH_4_^+^ but showed a sharp and highly significant increase to 84.12 μmol/g CDW at 75 mM NH_4_^+^ (*P* value ≤ 0.001) (Fig. 5; see also Data Set S5 at https://doi.org/10.6084/m9.figshare.20750203.v2). Concurrently, the intracellular concentration of ornithine was significantly increased and was greatest (167.20 μmol/g CDW) at 75 mM NH_4_^+^ (*P ≤ *0.001) (Fig. 5; see also Data Set S5 at https://doi.org/10.6084/m9.figshare.20750203.v2). Arginine and lysine were also most enriched at 75 mM NH_4_^+^, with 1,189.02 and 2,004.68 μmol/g CDW, respectively.

### NO_2_^−^ and N_2_O production.

**FIG 6:**
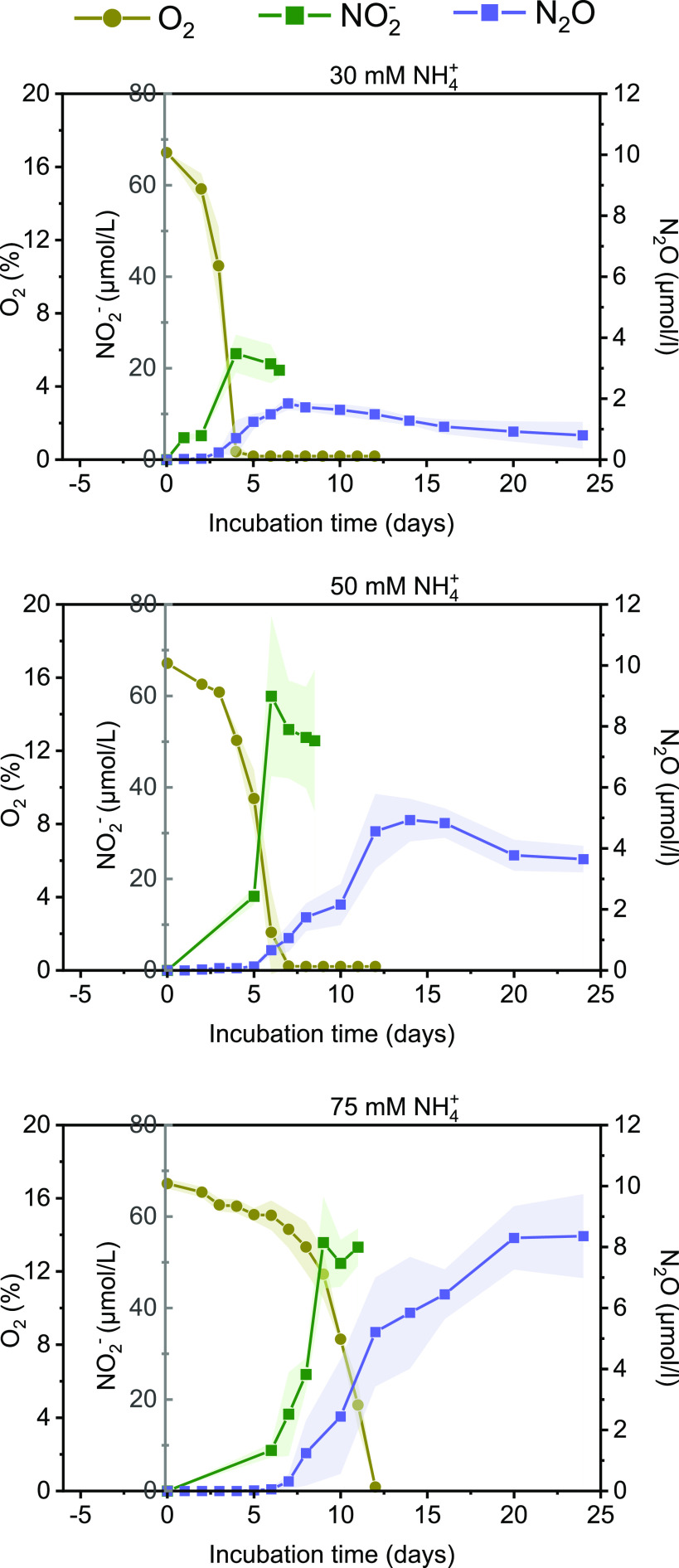
NO_2_^−^ and N_2_O production by *Methylocystis* sp. strain SC2 during exposure to 30 mM, 50 mM, and 75 mM NH_4_^+^. The shaded areas indicate the standard deviations of results of triplicate cultures. The amounts of NO_2_^−^ and N_2_O produced during growth with 1 mM and 10 mM NH_4_^+^ were negligible (see Fig. S8 at https://doi.org/10.6084/m9.figshare.20750236.v3, Table S3 at https://doi.org/10.6084/m9.figshare.20559417.v1, and Table S4 at https://doi.org/10.6084/m9.figshare.20589000.v1). While we measured the accumulation of N_2_O in the gaseous headspace, it needs to be noted that N_2_O is soluble in water at a ratio of 1:0.567 at 25°C (74).

NO_2_^−^ and N_2_O were detectable across all five NH_4_^+^ conditions (Fig. 6; see also Fig. S8 at https://doi.org/10.6084/m9.figshare.20750236.v3). The production of NO_2_^−^ significantly increased at 1 mM NH_4_^+^ to 50 mM NH_4_^+^ but did not further increase at 75 mM NH_4_^+^. The maximum concentrations of NO_2_^−^ that accumulated in the growth medium during the incubation experiments were 4.44 μmol/L (1 mM NH_4_^+^), 16.99 μmol/L (10 mM NH_4_^+^), 23.19 μmol/L (30 mM NH_4_^+^), 59.95 μmol/L (50 mM NH_4_^+^), and 54.32 μmol/L (75 mM NH_4_^+^) (Fig. 6; see also Fig. S8 at https://doi.org/10.6084/m9.figshare.20750236.v3 and Table S3 at https://doi.org/10.6084/m9.figshare.20559417.v1). The production of N_2_O significantly increased at 1 mM NH_4_^+^ to 75 mM NH_4_^+^. The maximum headspace concentrations of N_2_O that accumulated during the incubation experiments were 0.65 μmol/L (10 mM NH_4_^+^), 1.85 μmol/L (30 mM NH_4_^+^), 4.93 μmol/L (50 mM NH_4_^+^), and 5.84 μmol/L (75 mM NH_4_^+^) (Fig. 6; see also Fig. S8 at https://doi.org/10.6084/m9.figshare.20750236.v3 and Table S4 at https://doi.org/10.6084/m9.figshare.20589000.v1).

At high (50 mM and 75 mM) NH_4_^+^ levels, the NO_2_^−^ production rate was highly correlated with the greatest SC2 growth activity. Compared to NO_2_^−^ production, the accumulation of N_2_O was time shifted. Strong N_2_O accumulation occurred only after NO_2_^−^ had nearly reached its peak concentration (Fig. 6; see also Fig. S8 at https://doi.org/10.6084/m9.figshare.20750236.v3).

## DISCUSSION

In this study, we combined growth experiments with global proteomics, amino acid profiling, and nitrogen oxides measurements to thoroughly assess the response of *Methylocystis* sp. strain SC2 to increasing NH_4_^+^ concentrations. The ionic medium strength to which strain SC2 is able to acclimatize differs between NH_4_Cl and NaCl as the stressor. It is lower for NH_4_Cl (between 114 and 139 mM [present study]) than for NaCl (between 153 and 197 mM [31]). This may be due to the dual effect of an increasing NH_4_^+^ load, with one being a general stress phenomenon and another being the specific inhibition effect of ammonia on methanotrophic activity. Firstly, NH_4_Cl acted as an ionic and osmotic stressor, thereby leading to a tremendously increased lag phase duration with an increasing NH_4_^+^ load. Lag phase represents the earliest stage of the bacterial growth cycle and is defined by the adjustment of metabolic fluxes and enzyme composition to given environmental conditions (32, 33). The need for cellular adjustment processes directly depends on the level of environmental stress exposure. This view is congruous with our finding that the total number of differentially regulated proteins showed a highly positive and significant correlation with both lag phase duration and NH_4_^+^ load. Secondly, the CH_4_ consumption rate and, in consequence, the growth rate significantly decreased with the increasing NH_4_^+^ load due to the competitive inhibition of pMMO and the increasing need for detoxifying hydroxylamine, the product of pMMO-catalyzed oxidation of NH_3_. Notably, all three subunits of low-affinity pMMO1 were differentially regulated neither in response to increasing NH_4_^+^ load nor in response to high NaCl stress, regardless of whether the study was done on the transcriptome (31, 34) or the proteome (this study and reference 30) level. In the following, we first discuss the general stress response to increasing ionic and osmotic stress. Second, we discuss the methanotroph-specific response to hydroxylamine stress.

### General stress response to increasing ionic and osmotic stress.

The cellular adjustment processes in response to increasing ionic and osmotic stress are defined by proteomic rearrangements that are widely conserved among bacteria (35–37). These involve the upregulation of stress-responsive proteins, the K^+^ “salt-in” strategy, the uptake and/or synthesis of compatible solutes, and the induction of the glutathione metabolism pathway. The stress-responsive proteins upregulated in response to a high NH_4_^+^ load were the DNA-binding protein (Dps), the general stress response protein (CsbD), and heat shock proteins. Dps has a significant role in protecting the chromosome from oxidative damage but also from UV radiation, iron toxicity, heat, and pH stress (38). The protective stress-responsive function of CsbD family proteins is not yet known (39). While the expression of various heat shock proteins (e.g., Hsp10 [GroES], Hsp60 [GroEL], Hsp70 [DnaK], and Hsp100 [ClpB]) at a high constitutive level was not affected by NH_4_^+^, Hsp20 proteins were significantly enriched in response to a high NH_4_^+^ load. The Hsp20 machinery prevents aggregation and misfolding of client proteins and is known to be expressed upon exposure to a stressor (40–42).

In principle, two cellular strategies have evolved to cope with elevated osmolarity. The “salt-in” strategy leads to a rapid increase in the intracellular K^+^ pool, followed by a concomitant increase in the cytoplasmic concentration of compounds that are compatible with cell physiology at high internal concentrations. The uptake and/or synthesis of these compatible solutes or osmoprotectants is defined as a secondary response (36). Indeed, we observed a significant increase in the high-affinity (Kdp) K^+^ transport system under a high (75 mM) NH_4_^+^ load (Table 2). Concomitantly, global proteomics coupled with amino acid profiling revealed an intracellular glutamate pool that was significantly increased at high (50 mM and 75 mM) NH_4_^+^ levels (Fig. 5; Table 2). High glutamate concentrations are known to be required in a balanced osmoregulation to maintain a steady-state K^+^ pool (37, 43).

At a 75 mM NH_4_^+^ load, glutamate was replaced in part by proline to act as a compatible solute, again evidenced by global proteomics coupled with amino acid profiling. Acetylornithine aminotransferase, whose expression was significantly increased at a high NH_4_^+^ load, converts ornithine to Δ^1^-pyrroline-5-carboxylate, followed by the reduction to proline, with glutamate being the precursor for ornithine synthesis (44). Previous studies have shown that the cellular osmoadaptation gradually switches from potassium-glutamate as the dominant strategy at intermediate salinities to proline at higher salinities, with a 4- to 5-fold increase in the intracellular proline content (36, 45, 46). This is in the same range that we observed for the increase in intracellular proline content during the exposure of strain SC2 to a high (75 mM) NH_4_^+^ load. Thus, the intracellular accumulation of ornithine and proline under the maximum tolerable stress condition (75 mM NH_4_^+^) follows a widely distributed response pattern that is also known to occur in Escherichia coli and Bacillus subtilis (35, 47, 48).

The stress-triggered induction of the glutathione metabolism pathway involved a significant upregulation of both glutathione peroxidase (GPX) and glutathione *S*-transferase (GST) under a high NH_4_^+^ load. These enzymes have been shown to be expressed when cells are exposed to oxidative stress and hyperosmotic shock conditions. In particular, their activity is involved in detoxifying reactive oxygen (ROS) and nitrogen (RNS) species such as, for example, metal-bound NO^·^. The latter results in the formation of nitrosothiols (RSNO) and nitrosamines (RN_2_O), which are regarded as nonradical RNS (49). Notably, the expression of two GST isoforms (UniProt ID J7QHL1 and J7Q532) (Table 2) were specifically and significantly upregulated in response to an increasing NH_4_^+^ load. Their increase in expression level may have been induced by the increased production of both hydroxylamine (50) and RNS such as nitrite (19, 51).

Intriguingly, we also observed a differential regulation of various plasmid-encoded proteins, with most of them being upregulated (21 [pBSC2-1] and 18 [pBSC2-2]) (see Data Set S6 at https://doi.org/10.6084/m9.figshare.20750215.v2). On pBSC2-1, single-stranded DNA-binding protein (SSB), the three-component CzcCBA complex, and subunits of the F_o_F_1_ ATPase complex were among the proteins significantly upregulated under a high NH_4_^+^ load. SSB was the most greatly enriched (3.95-fold) plasmid-encoded protein during exposure of strain SC2 to 75 mM NH_4_^+^ (see Data Set S6 at https://doi.org/10.6084/m9.figshare.20750215.v2). It plays a major role in DNA replication, recombination, and repair. On pBSC2-2, multicopper oxidases and the type IV secretion system (T4SS) were among the proteins most significantly upregulated under a high NH_4_^+^ load, with the latter having functions in conjugation, DNA exchange with the extracellular space, and delivery of proteins to target cells (52). Significant enrichment of the pBSC2-2-encoded T4SS may be linked to the differential regulation of a PmoC subunit uniquely encoded by pBSC2-2. Moderately expressed under standard (1 and 10 mM) NH_4_^+^ growth conditions, this PmoC subunit showed the greatest downregulation (−4.8-fold) among all differentially regulated proteins in response to a high NH_4_^+^ load (Table 2), thereby providing further evidence for a particular cross talk between the SC2 chromosome and the two plasmids. Another major functional aspect is the location of various nitrogen-cycling genes on pBSC2-2 (discussed below).

More detailed information on the differential regulation of plasmid-encoded proteins in response to a high NH_4_^+^ load, but also on stress-responsive proteins, the K^+^ “salt-in” strategy, NH_4_^+^ assimilation, glutamate/glutamine metabolism, and glutathione metabolism, can be found in Text S2 in the supplemental material.

10.1128/msystems.00403-22.2 TEXT S2 Ammonium effects on the expression of stress-responsive proteins. Download Text S2, DOCX file, 0.09 MB. Copyright © 2022 Guo et al. 2022 Guo et al. https://creativecommons.org/licenses/by/4.0/ This content is distributed under the terms of the Creative Commons Attribution 4.0 International license.

### Methanotroph-specific response to hydroxylamine stress.

The apparent *K_m_* value for CH_4_ oxidation significantly increased with an increasing NH_4_^+^ load (Table 3), which is due to the increasing inhibition of pMMO-based CH_4_ oxidation by NH_3_. This inhibition effect was evident for 30 mM and 50 mM NH_4_^+^ but was most obvious for the CH_4_ consumption rate at 75 mM NH_4_^+^ (Fig. 2; Table 1). Unfortunately, the *K_m_*_(app)_ value could not be experimentally determined for the SC2 exposure to 75 mM NH_4_^+^ due to methodological constraints (Table 3). In addition, one may speculate that the increase in ionic and osmotic stress not only led to a prolonged duration of proteome adjustment but also had adverse effects on the CH_4_ oxidation activity of strain SC2.

**TABLE 3:** Effect of increasing NH_4_^+^ load on the apparent *K*_m_ and *V*_max_ values of CH_4_ oxidation^*a*^

CH_4_ (vol/vol, %)	NH_4_^+^ (mM)	*K_m_*_(app)_^*b*^ (μM)	*V*_max(app)_^*c*^ (mol cell^−1^ h^-1^)
2.5–20	10	0.17	2.96E−15
2.5–20	30	1.20**	2.32E−15
2.5–20	50	1.40**	2.07E−15

a CH_4_ and NH_4_^+^ constitute the incubation parameters.

b To test the inhibitory effect of increasing NH_4_^+^ concentrations on CH_4_ oxidation, SC2 cells were grown at 2.5%, 5%, 10%, 15%, and 20% CH_4_. When exposed to 75 mM NH_4_^+^, the growth of strain SC2 was completely inhibited when incubated with a headspace of 2.5% and 5% CH_4_. Therefore, the *K_m_*_(app)_ value for the treatment with 75 mM NH_4_^+^ could not be experimentally determined. Given the steady decline in the CH_4_ consumption rates (Table 1), it is, however, reasonable to conclude that at 75 mM NH_4_^+^, the *K_m_*_(app)_ value for CH_4_ oxidation was higher than it was for the incubation treatments with 30 mM and 50 mM NH_4_^+^. Multiplication with the Oswald constant (0.03395 at 25°C) gave the *K_m_*_(app)_ value for the methane concentration in water. The calculation of *K_m_*_(app)_ and *V*_max(app)_ values is based on triplicate cultures.

c The exponential decrease in CH_4_ over incubation time was used to estimate *V*_max(app)_ of SC2 cultures. Asterisks (**) indicate a significant difference (*P* value ≤ 0.01) relative to the 10 mM NH_4_^+^ condition.

Hydroxylamine is a highly toxic compound that has been shown to severely inhibit both calcium- and lanthanide-dependent methanol dehydrogenases (MDHs) (18, 53). This necessitates a rapid turnover of hydroxylamine in methanotrophic bacteria, which is most likely ensured by the activity of methanotrophic hydroxylamine oxidoreductase (mHAO). In strain SC2, both mHAO subunits (mHaoAB) showed a strong, significant upregulation concomitantly with the increase in NH_4_^+^ load (Table 2). This finding corroborates the conclusions lately drawn for the functional role of mHAO in the verrucomicrobial methanotroph Methylacidiphilum fumariolicum and other aerobic methanotrophs, namely, that mHAO plays a crucial role in preventing the inhibition of MDH (18). All subunits of the calcium-dependent Mxa-MDH (MxaFJGIRSACKLDH) were detectable in the SC2 proteome, with seven Mxa-MDH subunits (MxaFJCKLDH) being the only CH_4_ oxidation pathway proteins significantly enriched at a high (75 mM NH_4_^+^) load. The Mxa-MDH-associated cytochrome *c*_L_ (MxaG) was highly expressed constitutively. It is reasonable to assume that the significant enrichment of these seven Mxa-MDH subunits (including MxaF) is a proteomic response to compensate for the inhibitory effect of hydroxylamine. In contrast, the expression response of Xox-MDH varied, with XoxF being significantly downregulated at NH_4_^+^ loads of 30 and 50 mM (Table 2).

We observed a significant correspondence between the increase in NH_4_^+^ load and the accumulation of NO_2_^−^ and, with a delay, N_2_O (Fig. 6). This accumulation pattern has already been observed for a few proteobacterial methanotrophs in previous research, with the presumption that NO_2_^−^ is the final product of mHAO activity (54–57). However, recent purification of the mHAO from the verrucomicrobial methanotroph *Methylacidiphilum fumariolicum* provided biochemical evidence that this enzyme rapidly oxidizes hydroxylamine to NO rather than to NO_2_^−^. Conserved structural elements among all known mHAOs led to the further conclusion that this reaction mechanism occurs in all aerobic methanotrophs (18). Given that NO is an obligate free intermediate, one has to postulate either a yet unknown NO-oxidizing enzyme that converts NO to NO_2_^−^ or the spontaneous reaction with O_2_ to form NO_2_^−^ (18, 49, 58). Significant production of N_2_O occurred only after the oxygen concentration had dropped to low or unmeasurable levels (Fig. 6). This is in good agreement with previous reports that detoxification of hydroxylamine is directed toward increased production of N_2_O at hypoxic conditions (59, 60). Notably, the production of N_2_O from NO in strain SC2 does not involve the prior reduction of NO_2_^−^ to NO, because neither NirK nor NirS is encoded by its genome. This supports the conclusion of Versantvoort et al. (18) that NO is the end product of mHAO activity.

Candidate enzymes for the reduction of NO to N_2_O are a putative NO reductase (NorB) and hybrid cluster proteins (Hcps). NorB is encoded on pBSC2-2 but was not detectable in the SC2 proteome. Previous transcriptome research had shown, however, that relative to 10 mM NH_4_^+^, the transcript expression of the plasmid-borne *norB* significantly increased after a 10-h exposure of SC2 cells to 30 mM NH_4_^+^ (34). The inability to detect NorB in the SC2 proteome may be due to a large number of transmembrane domains, which makes it difficult to efficiently solubilize and digest NorB during the extraction of cellular proteins (61). The chromosome-encoded Hcp (UniProt ID J7Q787) is one of the most highly expressed proteins in strain SC2 and is significantly upregulated in response to increasing NH_4_^+^ levels (Table 2). Over the last decades, four different activities have been reported for Hcps (62). Among these is the activity as hydroxylamine reductase, which would lead to the production of NH_4_^+^/NH_3_ and thereby directly contribute to the detoxification of hydroxylamine. Being historically the first activity proposed (62), more recent research suggests, however, that the activity as hydroxylamine reductase has little or no physiological relevance. More likely is the conversion of NO to N_2_O (NO reductase activity), which has been established as physiologically relevant (62).

Notably, the pBSC2-2-encoded nitrous oxide reductase (NosZ) is constitutively expressed at a high level (see Data Set S6 at https://doi.org/10.6084/m9.figshare.20750215.v2), thereby suggesting that N_2_O may be further reduced to N_2_. The *nos* operon is located on a 20-kb region of pBSC2-2, which also contains the genes encoding NorB and two Hcp proteins (see Fig. S9 at https://doi.org/10.6084/m9.figshare.20750236.v3). This proximity of nitrogen-cycling genes (*norB*, *nosZ*), but also involving those encoding Hcps, further substantiates the functional relevance of pBSC2-2 for strain SC2. One of the two Hcps (UniProt ID I4EBE8) was also significantly enriched in response to an increasing NH_4_^+^ load, but its overall expression level was 1,000-fold lower than that of the chromosome-encoded Hcp protein (Table 2).

### Concluding remarks.

**FIG 7:**
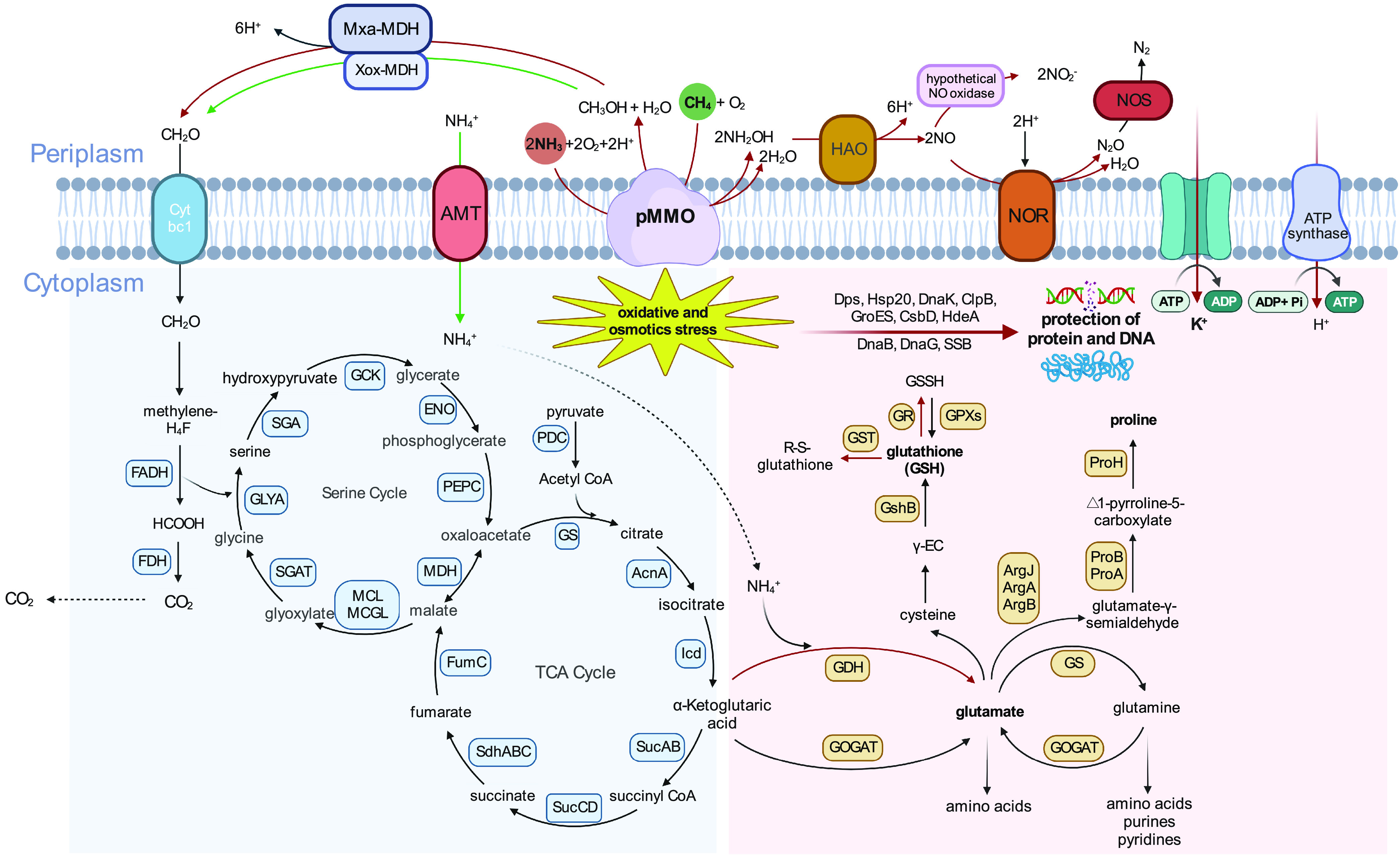
Scheme of the metabolic pathways and processes proposed to be involved in the acclimatization of *Methylocystis* sp. strain SC2 to a high NH_4_^+^ load. Proteins (enzymatic steps) and pathways that were significantly up- and downregulated are marked with red and green arrows, respectively. Black arrows indicate proteins (enzymatic steps) that were detectable in the proteome across all five NH_4_^+^ treatments but not differentially regulated. The response of strain SC2 to high (50 mM and 75 mM) NH_4_^+^ loads involved K^+^ influx (“salt-in” strategy) coupled to glutamate accumulation, in addition to increased production of various stress-responsive proteins. The intracellular accumulation of glutamate was achieved by a high expression level of glutamine synthetase and glutamate synthase (GS-GOGAT) and a significantly induced activity of glutamate dehydrogenase (GDH), which further fueled the biosynthesis of proline and other amino acids. Concomitantly, the synthesis of the ammonium transporter (Amt) and nitrogen regulatory protein P-II was significantly downregulated. After initiation of growth, the competitive interaction between CH_4_ and NH_3_ led to a significant increase in both the *K_m_*_(app)_ value for CH_4_ oxidation and the production of toxic hydroxylamine. Its detoxification involved the production and accumulation of nitrite (NO_2_^−^) and nitrous oxide (N_2_O) under a high NH_4_^+^ load, with NO as a putative intermediate (18). In addition, intermediates of reactive nitrogen species (RNS) may have triggered an antioxidant response involving the conversion of glutathione (GSH) into glutathione disulfide (GSSG) via the activity of glutathione peroxidase (GPX) and glutathione *S*-transferase (GST).

In this study, we comprehensively assessed the cellular ability of *Methylocystis* to acclimatize to a high NH_4_^+^ load. Our results provide detailed insights into how *Methylocystis* spp. adjust their cells to cope with the dual effect of NH_4_^+^, namely, ionic and osmotic stress and competitive interaction between CH_4_ and NH_3_ (Fig. 7). Indeed, our results show that *Methylocystis* has the capacity to precisely acclimatize to changes in NH_4_^+^ concentration by exact physiological rebalancing enzymes and osmolyte composition, thereby enabling maintenance of a suitable cellular homeostasis for growth. The maximum NH_4_^+^ tolerance of *Methylocystis* sp. strain SC2 (75 mM NH_4_^+^) was in the same range as previously shown for Methylosinus sporium (71 mM NH_4_^+^) (55). The need to simultaneously combat both ionic-osmotic stress and the toxic effects of hydroxylamine and nitrite is presumably the limiting factor for the cellular acclimatization of *Methylocystis* spp. to higher NH_4_^+^ concentrations (Fig. 7).

## MATERIALS AND METHODS

### Strain.

The genome of *Methylocystis* sp. strain SC2 was found to comprise a 3.77-Mb chromosome and two large plasmids (63, 64). Their nucleotide sequences are publicly available in EMBL, GenBank, and DDBJ databases under accession numbers HE956757 (chromosome) and FO000001 and FO000002 (plasmids). Genomic analysis revealed the presence of a complete denitrification pathway in strain SC2 (65). Strain SC2 has the ability to produce low- and high-affinity pMMO isozymes and can thus oxidize CH_4_ across a wide concentration range (6, 66). The low-affinity pMMO1 is encoded by two *pmoCAB1* gene clusters, while the high-affinity pMMO2 is encoded by a single *pmoCAB2* gene cluster (64). In addition, the genome of strain SC2 contains two chromosome-encoded monocistronic *pmoC* genes (*pmoC1_Gs_*, *pmoC2_Gs_*) and a single plasmid-borne *pmoC* gene (*pmoC_Ps_*) (63).

### Experimental procedures.

Strain SC2 cells were first inoculated into 40 mL nitrate-containing mineral salts (NMS) medium in 120-mL serum bottles and grown to an OD_600_ of 0.25 ± 0.05 (Fig. 1). The composition of NMS growth medium was the same as previously reported (67), containing 1 g of KNO_3_ per L as the nitrogen source. Strain SC2 was precultured in NMS medium at least twice and then used to investigate the effect of increasing ammonium concentrations in mineral salts (AMS) medium on its cell density, CH_4_ consumption, and CO_2_ production. A 1-mL aliquot of NMS-precultured SC2 cells was inoculated into 120-mL serum bottles containing 40 mL AMS medium. The initial OD_600_ was 0.01 ± 0.003. The composition of AMS was the same as that of NMS, with the exception that 1 g of KNO_3_ (10 mM) was replaced by increasing amounts of NH_4_Cl. This resulted in treatment concentrations of 1, 10, 30, 50, 75, and 100 mM NH_4_Cl in the medium (Fig. 1), corresponding to a total ionic strength ranging from 40 mM (1 mM NH_4_Cl) to 139 mM (100 mM NH_4_Cl) (see Table S2 in the supplemental material). The headspace of the batch cultures was filled with filter (0.20-μm-pore-size)-sterilized CH_4_ and air at a 20:80 (vol/vol) ratio. The serum bottles were sealed with rubber stoppers and incubated on a rotary shaker at 130 rpm and 25°C. Both OD_600_ and changes in the headspace concentrations of CH_4_ and CO_2_ were regularly monitored during the whole incubation period (Fig. 1).

### Physiological parameters.

The OD_600_ was determined using an Eppendorf BioPhotometer UV/Vis spectrophotometer (Eppendorf, Germany). Cell dry weight (CDW) was calculated based on the following relationship: biomass (g CDW) = OD_600_ × 0.261 × volume (68). Biomass yield is shown as milligrams of CDW/mmol of CH_4_. Methane consumption and CO_2_ production were analyzed by gas chromatography (SRI Instruments, Torrance, CA). The methane consumption rate is indicated as millimoles of CH_4_ consumed/g of CDW/day. All rate calculations are based on parameter values measured during exponential growth. The production of N_2_O was monitored using an N_2_O microsensor with a piercing needle. The microsensor was connected to a microsensor multimeter (Unisens A/S, Denmark). The O_2_ concentration in the headspace was monitored with a Fibox 4 trace meter using SP-PSt3 sensor spots. This yielded an oxygen detection limit as low as 0.002% (by volume) (PreSens; https://www.presens.de/). The production of NO_2_^−^ was determined using the Griess reagent system by following the manufacturer`s instruction (Promega Corporation, Madison, WI).

### Methane oxidation kinetics [*K_m_*_(app)_ and *V*_max(app)_] calculations.

To test for the inhibitory effect of NH_4_^+^ on CH_4_ oxidation, SC2 cells were grown at the following CH_4_-air mixing ratios: 20:80, 15:85, 10:90, 5:95, and 2.5:97.5 (vol/vol). Each CH_4_-air mixing ratio was tested in triplicate incubations under three different ammonium concentrations (10 mM, 30 mM, and 50 mM NH_4_^+^). Cell density (OD_600_) and CH_4_ concentration in the headspace were regularly measured over the whole incubation period. Cell densities were converted into cell numbers as described previously (6). An OD_600_ value of 1 corresponds to about 1.5 × 10^8^ cells mL^−1^ in the exponential growth phase. The exponential decrease of CH_4_ over incubation time was used to estimate *K_m_*_(app)_ and the maximum apparent rate of metabolism [*V*_max(app)_] of SC2 cultures using nonlinear regression with the Michaelis-Menten equation. Multiplication by the Oswald constant (0.03395 at 25°C) gave the *K_m_*_(app)_ as the methane concentration in water (6, 69).

### Sample preparation for proteomics.

Samples for proteomics were collected from the same cultures. Strain SC2 was inoculated into 300 mL mineral salts medium (initial OD_600_ of 0.01 ± 0.003) supplemented with 1, 10, 30, 50, or 75 mM NH_4_^+^ (Fig. 1). Cells were grown to the mid-exponential phase (OD_600_ = 0.25 ± 0.02) and then collected by centrifugation at 7,000 × *g* and 4°C for 20 min. The cells were thoroughly washed twice with 1× phosphate buffer (5.4 g Na_2_HPO_4_ · 7 H_2_O and 2.6 g KH_2_PO_4_ per L of distilled H_2_O) to remove medium traces. The washed cell pellets were transferred to 2 mL sterile Safe-Lock microcentrifuge tubes (Eppendorf) and stored at –80°C for subsequent protein extraction. Each NH_4_^+^ concentration involved the analysis of triplicate cultures.

### Protein extraction, LC-MS/MS analyses, peptide/protein identification, and LFQ quantification.

The extraction of the total SC2 proteins was done as described previously, using an efficient tandem LysC-trypsin digestion in a detergent condition (30). The liquid chromatography-tandem mass spectrometry (LC-MS/MS) analysis of protein digests was performed on a Q-Exactive Plus mass spectrometer connected to an electrospray ion (ESI) source (Thermo Fisher Scientific). Peptide separation was carried out using the UltiMate 3000 RSLCnanoLC system (Thermo Fisher Scientific) equipped with an in-house packed C_18_ resin column (Magic C_18_ AQ 2.4 μm; Dr. Maisch). The peptides were first loaded onto a C_18_ precolumn (preconcentration setup) and then eluted in backflush mode using a gradient from 96% solvent A (0.15% formic acid) and 4% solvent B (99.85% acetonitrile, 0.15% formic acid) to 30% solvent B over 115 min. The flow rate was set to 300 nL/min. The data acquisition mode for the initial label-free quantification (LFQ) study was set to obtain one high-resolution MS scan at a resolution of 60,000 (*m/z* 200) with a scanning range from 375 to 1,500 *m/z*, followed by MS/MS scans of the 10 most intense ions. To increase the efficiency of MS/MS acquisition, the charged-state screening modus was activated to exclude unassigned and singly charged ions. The dynamic exclusion duration was set to 30 s. The ion accumulation time was set to 50 ms (both MS and MS/MS). The automatic gain control (AGC) was set to 3 × 10^6^ for MS survey scans and 1 × 10^5^ for MS/MS scans (for details, see reference 30).

### Statistical and functional analysis of differentially regulated proteins.

Discovery-LFQ was done using Progenesis QI software (Nonlinear Dynamics, version 2.0) as described before (for details, see reference 30). Next, the data obtained from Progenesis were evaluated using SafeQuant R package, version 2.2.2 (70). Hereby, a 1% identification and quantification false discovery rate (FDR) were calculated. Differentially regulated proteins (DRPs) with a log_2_ fold change greater than or equal to 1 (upregulated) or less than or equal to −1 (downregulated) and a *q* value of  ≤0.01 were submitted to the Kyoto Encyclopedia of Genes and Genomes (KEGG) database for enrichment function analysis.

### Sampling and extraction of intracellular metabolites.

SC2 cells were grown to mid-exponential phase in 120-mL serum bottles containing 40 mL mineral salts medium supplemented with 1 mM, 10 mM, 30 mM, 50 mM, and 75 mM NH_4_^+^ (Fig. 1). Aliquots (36 mL) of 60% (vol/vol) methanol in a 50-mL conical centrifuge tube were cooled down to −80°C for 48 h and then used as quenching solution. Twelve-milliliter culture aliquots (*n* = 4) were pipetted into the quenching solution, and the quenched cells were immediately pelleted in an Eppendorf 5430R centrifuge for 10 min at 10,000 × *g* and −10°C, using a fixed-angle rotor. After centrifugation, the supernatant was removed and the cell pellets were stored at −80°C until further extraction of the endometabolome.

The endometabolome was extracted by suspending the frozen cell pellets in equal volumes of extraction fluid (−20°C) and chloroform (−20°C). The extraction volume was adapted to sample biomass, using 1 mL of extraction fluid and an equal volume of chloroform per 1 mL of sample at an OD_600_ of 1. The extraction fluid consisted of 50% (vol/vol) methanol at LC-MS grade and 50% (vol/vol) TE buffer (10 mM Trizma, 1 mM EDTA). The resulting cell suspension was incubated in a ThermoMixer C shaker (Eppendorf) at 4°C for 2 h (1,500 rpm), followed by a two-phase separation of the suspension in an Eppendorf 5430R centrifuge for 10 min at 12,000 × *g* and −10°C, using a fixed-angle rotor. The upper phase was filtered through a 0.20-μm polytetrafluoroethylene (PTFE) membrane filter (Phenomenex) into 2-mL sterile Safe-Lock microcentrifuge tubes (Eppendorf). The metabolite extracts were stored at −80°C until downstream analysis.

### Measurement of amino acids.

Quantitative determination of amino acids was performed using LC-MS/MS. The chromatographic separation was performed on an Agilent Infinity II 1260 high-performance liquid chromatography (HPLC) system using a SeQuant ZIC-HILIC column (150 by 2.1 mm, 3.5-μm particle size, 100-Å pore size) connected to a ZIC-HILIC guard column (20 by 2.1 mm, 5-μm particle size) (Merck KGaA), with a constant flow rate of 0.3 mL/min with mobile phase A being 0.1% formic acid in 99:1 water-acetonitrile (Honeywell, Morristown, NJ, USA) and phase B being 0.1% formic acid in 99:1 water-acetonitrile (Honeywell, Morristown, NJ, USA) at 25°C.

The injection volume was 1 μL. The mobile phase profile consisted of the following steps and linear gradients: 0 to 8 min from 80% to 60% B; 8 to 10 min from 60% to 10% B; 10 to 12 min constant at 10% B; 12 to 12.1 min from 10% to 80% B; 12.1 to 14 min constant at 80% B. An Agilent 6470 mass spectrometer was used in positive mode with an ESI source and the following conditions: ESI spray voltage of 4,500 V, nozzle voltage of 1,500 V, sheath gas of 400°C at 12 L/min, nebulizer pressure of 30 lb/in^2^, and drying gas of 250°C at 11 L/min. Compounds were identified based on their mass transition and retention times in comparison to standards. Chromatograms were integrated using MassHunter software (Agilent, Santa Clara, CA, USA). Absolute concentrations were calculated based on an external calibration curve prepared in a sample matrix.

### Computational analysis.

Hierarchical heat map analysis was performed on Z-score-normalized LFQ intensities of the total of 438 DRPs. Creation of both the hierarchical heat map and the Venn diagram, but also performance of the PCA, was done using the free online platform for data analysis and visualization available at https://www.bioinformatics.com.cn/. The volcano plots were created using VolcaNoseR (71). The STRING database was used to construct the PPI network based on the Uniprot IDs of the total DRPs, thereby resulting in automated calculation of edges and nodes using the default value for the minimum interaction score (0.4). Gephi (version 0.9.2), an open-source software, was used for modularity calculation and visualization (72). Nodes with no or less than four edges were omitted, thereby resulting in a PPI network of 121 nodes (proteins) that are connected by a total of 431 edges. The final presentation layout of the PPI network was created with Fruchterman Reingold, a method implemented in Gephi.

### Software used for preparation of figures and graphs.

Figures and graphs were created with (i) Sigmaplot version 14.0, (ii) OriginPro 2020, (iii) GraphPad Prism 9.0.2, (iv) commercial software TIBCO Spotfire, and (v) and Adobe Illustrator 2020.

### Data availability.

Various supplemental figures (S1 to S9), tables (S3 and S4), and data sets (S2 to S6) are available at https://figshare.com/projects/Methylocystis_sp_Strain_SC2_Acclimatizes_to_Increasing_NH4_Levels_by_a_Precise_Rebalancing_of_Enzymes_and_Osmolyte_Composition_-_supplementary_files/147147. The MS proteomics data have been deposited with the ProteomeXchange Consortium via the PRIDE (73) partner repository under the data set identifier PXD032347.
