# Supplementary material for: Methylocystis sp. Strain SC2 Acclimatizes to Increasing NH4+ Levels by a Precise Rebalancing of Enzymes and Osmolyte Composition
Source: mSystems. 2022 Sep 26;7(5):e00403-22. doi: 10.1128/msystems.00403-22 (PMC9600857; doi:10.1128/msystems.00403-22)
Supplement: TEXT S1 [file msystems.00403-22-s0001.docx]

**Appendix S1: Supplemental Introduction**

***Environmental effects of ammonium on the methanotrophic activity***

Aerobic methane-oxidizing bacteria, or methanotrophs, attenuate CH_4_ emission from major sources (e.g., natural wetlands, rice paddies, landfills) and constitute the only biological sink for atmospheric CH_4_ in upland soils. Their activity in soil is highly sensitive to the nitrogen source. Ammonia concentrations in the lower millimolar range have been reported to partially or completely inhibit CH_4_ oxidation (1-3). Whether NH_4_^+^ in the environment has inhibitory or stimulatory effects on methane-oxidizing bacteria, however, depends largely on the diversity, structure and activity of the methanotrophic community, as well as the particular conditions in the habitat (4-7). The application of NH_4_^+^ fertilizers to various soils and sediments has been shown to inhibit methanotrophic activity (8, 9). Long-term effects were observed particularly for atmospheric CH_4_ oxidation in various upland soils (9, 10). The key players of CH_4_ methane oxidation are thought to be poorly characterized methanotrophs named Upland Soil Cluster (USC) α and γ (11-15). *Methylocystis* spp., however, are also widely distributed in upland and hydromorphic soils and presumably make a major contribution to the oxidation of atmospheric methane (12, 16-18). Indeed, recent research has unambiguously shown that *Methylocystis* spp. contribute via the expression of their high-affinity pMMO2 (19) to the atmospheric CH_4_ sink in grasslands (20).

On the other hand, methanotrophs, like all bacteria, require nitrogen for growth. Therefore, contrary to the inhibitory effects observed, NH_4_^+^ fertilization may stimulate CH_4_ oxidation activity in some cases (9, 21). For example, methanotrophic activity and growth were found to be stimulated after CH_4_ fertilization in the root zone of rice plants (22, 23). Later studies suggested that nitrogenous fertilizers stimulate consumption of CH_4_ and subsequent growth of many, but not all, gammaproteobacterial type I methanotrophs. By contrast, the activity of alphaproteobacterial type II methanotrophs (*Methylocystis* / *Methylosinus*) is inhibited or at least not stimulated in nitrogen-amended soils (24, 25).

Another example are termites that are known to be a major source of CH_4_ but not to contain methanotrophic activity (26). It has been assumed that, among other reasons, high NH_3_ concentration may be responsible for the absence of CH_4_ oxidation in termite hindguts (27).

Thus, besides CH_4_, NH_3_ is a key factor determining methanotrophic activity in the environment. The particulate methane monooxygenase (pMMO) is evolutionarily related to the ammonia monooxygenase (AMO) (28). The structural homology between pMMO and AMO allows both methanotrophs and ammonia oxidizers to convert either substrates (CH_4_ and NH_3_), although neither is able to grow on the alternative substrate (29-31). The pMMO oxidizes NH_3_ to hydroxylamine (NH_2_OH) (30, 31). Ammonia produced from the deprotonation of liquid NH_4_^+^ competes with CH_4_ for the same active site of pMMO (25). Because NH_2_OH is a highly toxic intermediate, methanotrophs rely on the ability to remove it quickly (30). The fact that NH_3_ acts for methanotrophs as both a nutrient and a competitive inhibitor of pMMO suggests that these bacteria need to be able to simultaneously acclimatize to various environmental triggers: assimilation of NH_4_^+^ nitrogen, competitive inhibition of pMMO, and NH_2_OH and NO_2_^-^ toxicity when NH_3_ oxidation is favored (30).

**References**

1. Bosse U, Frenzel P, Conrad R. 1993. Inhibition of methane oxidation by ammonium in the surface layer of a littoral sediment. FEMS Microbiol Ecol 13:123–134.
2. King GM, Schnell S. 1998. Effects of ammonium and non-ammonium salt additions on methane oxidation by *Methylosinus trichosporium* OB3b and Maine forest soils. Appl Environ Microbiol 64:253–257.
3. Conrad R, Rothfuss F. 1991. Methane oxidation in the soil surface layer of a flooded rice field and the effect of ammonium. Biol Fertil Soils 12:28-32.
4. Stein LY, Roy R, Dunfield PF. Aerobic Methanotrophy and Nitrification: Processes and Connections, eLS doi:<https://doi.org/10.1002/9780470015902.a0022213>.
5. Dunfield P, Knowles R. 1995. Kinetics of inhibition of methane oxidation by nitrate, nitrite, and ammonium in a humisol. Appl Environ Microb 61:3129-3135.
6. Mohanty SR, Bodelier PLE, Floris V, Conrad R. 2006. Differential Effects of Nitrogenous Fertilizers on Methane-Consuming Microbes in Rice Field and Forest Soils. Appl Environ Microb 72:1346-1354.
7. Schnell S, King GM. 1994. Mechanistic Analysis of Ammonium Inhibition of Atmospheric Methane Consumption in Forest Soils. Appl Environ Microb 60:3514-3521.
8. Van Der Nat F, De Brouwer J, Middelburg JJ, Laanbroek HJ. 1997. Spatial distribution and inhibition by ammonium of methane oxidation in intertidal freshwater marshes. Appl Environ Microbiol 63:4734–4740.
9. Bodelier PL, Laanbroek HJ. 2004. Nitrogen as a regulatory factor of methane oxidation in soils and sediments. FEMS Microbiol Ecol 47**:**265–277.
10. Gulledge J, Schimel JP. 1998. Low-concentration kinetics of atmospheric CH_4_ oxidation in soil and mechanism of NH_4_^+^ inhibition. Appl Environ Microbiol 64:4291–4298.
11. Knief C, Lipski A, Dunfield PF. 2003. Diversity and activity of methanotrophic bacteria in different upland soils. Appl Environ Microbiol 69:6703–6714.
12. Dunfield PF. 2007. The soil methane sink. In *Greenhouse Gas Sinks*. Reay, D., Hewitt, C.N., Smith, K., and Grace, J. (eds). Wallingford, UK: CABI Publishing, pp. 152–170.
13. Shrestha PM, Kammann C, Lenhart K, Dam B, Liesack Werner. 2012. Linking activity, composition and seasonal dynamics of atmospheric methane oxidizers in a meadow soil. ISMEJ 6:1115-1126.
14. Knief C. 2015. Diversity and habitat preferences of cultivated and uncultivated aerobic methanotrophic bacteria evaluated based on *pmoA* as molecular marker. Front Microbiol 6:e1346.
15. [Pratscher](https://sfamjournals.onlinelibrary.wiley.com/action/doSearch?ContribAuthorRaw=Pratscher%2C+Jennifer) J, [Vollmers](https://sfamjournals.onlinelibrary.wiley.com/action/doSearch?ContribAuthorRaw=Vollmers%2C+John) J, [Wiegand](https://sfamjournals.onlinelibrary.wiley.com/action/doSearch?ContribAuthorRaw=Wiegand%2C+Sandra) S, [Dumont](https://sfamjournals.onlinelibrary.wiley.com/action/doSearch?ContribAuthorRaw=Dumont%2C+Marc+G) MG, Kaster A-K. 2018. Unravelling the identity, metabolic potential and global biogeography of the atmospheric methane-oxidizing upland soil cluster α. Environ Microbiol 20:1016-1029.
16. Knief C, Dunfield PF. 2005. Response and adaptation of different methanotrophic bacteria to low methane mixing ratios. Environ Microbiol **7:**1307–1317.
17. Knief C, Vanitchung S, Harvey NW, Conrad R, Dunfield PF, Chidthaisong A. 2005. Diversity of methanotrophic bacteria in tropical upland soils under different land uses. *A*ppl Environ Microbiol 71:3826–3831.
18. Knief C, Kolb S, Bodelier PL, Lipski A, Dunfield PF. 2006. The active methanotrophic community in hydromorphic soils changes in response to changing methane concentration. Environ Microbiol 8:321–333.
19. Baani M, Liesack W. 2008. Two isozymes of particulate methane monooxygenase with different methane oxidation kinetics are found in *Methylocystis* sp. strain SC2. PNAS 105: 10203–10208.
20. Täumer J, Marhan S, [Groß](https://pubmed.ncbi.nlm.nih.gov/?term=Gro%C3%9F+V&cauthor_id=35388141) V, [Jensen](https://pubmed.ncbi.nlm.nih.gov/?term=Jensen+C&cauthor_id=35388141) C, [Kuss](https://pubmed.ncbi.nlm.nih.gov/?term=Kuss+AW&cauthor_id=35388141) AW, [Kolb](https://pubmed.ncbi.nlm.nih.gov/?term=Kolb+S&cauthor_id=35388141) S, Urich T. 2022. Linking transcriptional dynamics of CH _4_-cycling grassland soil microbiomes to seasonal gas fluxes. ISMEJ 16:1788-1797.
21. Stein LY, Roy R, Dunfield PF. 2012. Aerobic methanotrophy and nitrification: processes and connections. In Encyclopedia of Life Sciences. Battista, J., *et al*. (ed.). Chichester, UK: John Wiley & Sons [WWW document]. URL <http://www.els.net>.
22. Bodelier PL, Roslev P, Henckel T, Frenzel P. 2000. Stimulation by ammonium-based fertilizers of methane oxidation in soil around rice roots. Nature 403:421–424.
23. Krüger M, Frenzel P. 2003. Effects of N-fertilisation on CH4 oxidation and production, and consequences for CH4 emissions from microcosms and rice fields. Glob Change Biol 9:773–784.
24. Mohanty SR, Bodelier PL, Floris V, Conrad R. 2006. Differential effects of nitrogenous fertilizers on methane-consuming microbes in rice field and forest soils. Appl Environ Microbiol 72:1346–1354.
25. Nyerges G, Stein LY. 2009. Ammonia cometabolism and product inhibition vary considerably among species of methanotrophic bacteria. FEMS Microbiol Lett 297:131–136.
26. Brauman A, Kane MD, Labat M, Breznak JA. 1992. Genesis of acetate and methane by gut bacteria of nutritionally diverse termites. Science 257:1384–1387.
27. Pester M, Tholen A, Friedrich MW, Brune A. 2007. Methane oxidation in termite hindguts: Absence of evidence and evidence of absence. Appl Environ Microbiol 73:2024-2028.
28. Holmes AJ, Costello A, Lidstrom ME, Murrell JC. 1995. Evidence that particulate methane monooxygenase and ammonia monooxygenase may be evolutionarily related. FEMS Microbiol Lett 132: 203–208.
29. Bédard C, Knowles R. 1989. Physiology, biochemistry, and specific inhibitors of CH_4_, NH_4_^+^, and CO oxidation by methanotrophs and nitrifiers. Microbiol Rev 53:68-84.
30. Stein LY, Klotz MG. 2011. Nitrifying and denitrifying pathways of methanotrophic bacteria. Biochem Soc Trans 39:1826-1831.
31. Versantvoort W, Pol A, Jetten MSM, Niftrik Lv, Reimann J, Kartal B, Camp HJMOd. 2020. Multiheme hydroxylamine oxidoreductases produce NO during ammonia oxidation in methanotrophs. PNAS 117:24459-24463.
