# Supplementary material for: Methylocystis sp. Strain SC2 Acclimatizes to Increasing NH4+ Levels by a Precise Rebalancing of Enzymes and Osmolyte Composition
Source: mSystems. 2022 Sep 26;7(5):e00403-22. doi: 10.1128/msystems.00403-22 (PMC9600857; doi:10.1128/msystems.00403-22)
Supplement: TABLE S2 [file msystems.00403-22-st002.pdf]

Table S2. Growth medium: ionic strength, pH, dissolved CH_4, aqueous NH_3, and CH_4/NH_3 ratio across the NH_4Cl treatments.

	Ionic strength (IS, mM)				pH	Aqueous CH_4 (mM)[1]	Aqueous NH_3 (mM)[2]	CH_4/NH_3
	Trace element + phosphate buffer	NH_4^+	Cl^-	Total (IS)				
1 mM AMS	39.3	0.5	0.5	40.4	7.22	0.27	8.94E-03	30.63
10 mM AMS	39.3	5.0	5.0	49.4	7.11	0.27	7.11E-02	3.85
30 mM AMS	39.3	15.1	15.1	69.6	7.04	0.27	1.70E-01	1.61
50 mM AMS	39.3	25.2	25.2	89.8	7.00	0.27	2.83E-01	0.97
75 mM AMS	39.3	37.9	37.9	115.1	6.95	0.27	3.38E-01	0.81
100 mM AMS	39.3	50.5	50.5	140.3	-		-	

[1]Dissolved CH_4 was calulated using the Henry coefficient as proposed by Serra et al. (2006) for CH_4 solubility in methanotroph growth media: Serra MC, Pessoa FLP, Palavra AMF. 2006. Solubility of methane in water and in the medium for the cultivation of methanotrophs bacteria. J Chem Thermodynamics 38:1629-1633.

[2]The aqueous NH_3 concentrations in the growth medium was estimated according to Thurston GD, et. al. (1979): Aqueous Ammonia Equilibrium-Tabulation of Percent Un-ionized Ammonia. EPA-600/3-79-091, Environmental Research Laboratory, Duluth, MN 55804. Available from NTIS (PB80-103518).
