# Supplementary material for: Methylocystis sp. Strain SC2 Acclimatizes to Increasing NH4+ Levels by a Precise Rebalancing of Enzymes and Osmolyte Composition
Source: mSystems. 2022 Sep 26;7(5):e00403-22. doi: 10.1128/msystems.00403-22 (PMC9600857; doi:10.1128/msystems.00403-22)
Supplement: TABLE S1 [file msystems.00403-22-st001.pdf]

TABLE S1 Physiological parameter of *Methylocystis* sp. strain SC2 measured in response to various CH_4-air mixing ratios under increasing NH_4^+ concentrations (see Fig. S1 at https://doi.org/10.6084/m9.figshare.20750236.v3 for a graphic presentation of the physiological growth parameters).

NH_4^+ conc. (mM)	CH_4 conc. (%)	CDW (mg)	Growth rate (mg CDW/d)	CH_4 consumption (mmol CH_4)	Biomass yield (mg CDW/mmol CH_4)	CH_4 consumption rate (mmol CH_4/g CDW/d)
**10 mM NH_4^+**	**20%**	3.89±0.36	1.94±0.18	0.32±0.02	12.23±1.88	41.58±6.98
	**15%**	3.71±0.39	1.85±0.2	0.30±0.02	12.36±2.27	40.03±9.32
	**10%**	3.47±0.1	1.74±0.05	0.24±0.01***	14.48±0.3	34.54±0.72
	**5%**	1.60±0.02***	0.80±0.01***	0.11±0.00***	14.75±0.75	33.96±1.75
	**2.5%**	0.67±0.08***	0.33±0.04***	0.05±0.00***	14.00±1.77	36.09±4.58
**30 mM NH_4^+**	**20%**	3.56±0.14	1.19±0.05	0.28±0.08	13.46±4.49	26.42±7.47
	**15%**	3.66±0.28	1.22±0.09	0.32±0.03	11.56±1.66	29.28±4.58
	**10%**	3.54±0.08	1.18±0.03	0.21±0.04	17.13±3.60	20.00±3.90
	**5%**	1.93±0.09***	0.64±0.03***	0.11±0.00	17.98±1.47*	18.63±1.60
	**2.5%**	1.02±0.08***	0.34±0.03***	0.04±0.00	24.09±2.05**	13.90±1.17*
**50 mM NH_4^+**	**20%**	3.06±0.28	0.51±0.05	0.42±0.08	7.53±1.96	27.90±7.86
	**15%**	3.28±0.40	0.55±0.07	0.37±0.02	9.00±1.16	22.47±2.98
	**10%**	2.80±0.23	0.47±0.04	0.30±0.01	9.30±0.66	21.58±1.47
	**5%**	0.09±0.05***	0.02±0.01***	0.05±0.01***	1.97±0.59***	BDL
	**2.5%**	BDL	BDL	0.04±0.00***	BDL	BDL

Note: BDL, below detection level. Significant difference is calculated in relation to the control treatment (1 mM NH_4^+) using one-way ANOVA Bonferroni t-test: *p-value≤0.05; **p-value≤0.01; ***p-value≤0.001.
