# Supplementary material for: Methylocystis sp. Strain SC2 Acclimatizes to Increasing NH4+ Levels by a Precise Rebalancing of Enzymes and Osmolyte Composition
Source: mSystems. 2022 Sep 26;7(5):e00403-22. doi: 10.1128/msystems.00403-22 (PMC9600857; doi:10.1128/msystems.00403-22)
Supplement: TEXT S2 [file msystems.00403-22-s0002.docx]

**Appendix S2: Supplemental Discussion**

***Ammonium effects on the expression of stress-responsive proteins***

**Dps.** The DNA-binding protein (Dps) is widely distributed among bacteria (e.g., *Escherichia coli*, *Bacillus subtilis*, *Thermosynechococcus elongatus*), and its vital role in contributing to stress survival is remarkably conserved (1-3). In strain SC2, the expression of Dps was significantly induced upon exposure to high NH_4_^+^ levels. Dps has a significant structural homology to ferritins and possesses the ability to sequester iron and ferroxidase activity (4). Its iron-binding activity plays a significant role in protecting the chromosome from oxidative damage during exponential growth of *E. coli*, but Dps has been shown to also protect *E. coli* cells during the stationary growth phase against UV, iron toxicity, heat and pH stress (4).

**CsbD**. CsbD is a bacterial general stress response protein. Its expression is mediated by sigma-B, an alternative sigma factor (5). However, the role of CsbD in stress response remains elusive. In strain SC2, two CsbD family proteins and, in agreement with literature data, one sigma factor of CsbD (RpoH [σ^32^]) were co-upregulated under high NH_4_^+^ load. Sigma factor RpoH is the key regulator of the heat shock response in *E. coli* (6).

**Heat shock response system.** In strain SC2, global proteomics identified the expression of the following heat shock response (HSR) systems: Hsp10 (GroES), Hsp20, Hsp60 (GroEL), Hsp70 (DnaK/DnaJ/GrpE), Hsp90, and Hsp100 (ClpB). The expression of Hsp20, also known as small heat shock proteins (sHSPs), was significantly upregulated in response to high NH_4_^+^ load (Table 3). sHSPs play an important role in maintaining cell proteostasis. Their most widespread and evolutionarily conserved function is binding to denaturing polypeptides, thereby acting as protein chaperones. Indeed, sHSPs serve as an efficient binding reservoir for unstable protein-folding intermediates during stress. Once cells are exposed to sudden proteotoxic stress, Hsp20s encage and shield accumulated aggregation-prone nonnative proteins to ensure their correct folding in near-native conformations. Refolding occurs via the activity of the Hsp70 and Hsp100 systems as soon as environmental conditions become favorable. The assemblies are recognized by Hsp70 and further processed by both Hsp70 and Hsp100, which leads in an ATPase-dependent manner to the extraction of single polypeptides from aggregates and their subsequent refolding (7). In strain SC2, none of the Hsp70 (DnaK/DnaJ/GrpE) and Hsp100 (ClpB) proteins showed a differential expression response to increasing NH_4_^+^ load. However, DnaK (Hsp70) and Hsp100 (ClpB) were constitutively expressed at high level. In summary, while the refolding machinery is constitutively expressed in SC2 cells, the Hsp20 machinery to prevent aggregation and misfolding of client proteins is expressed only upon exposure to a stressor such as high NH_4_^+^ load (Table 3). Hsp60 (GroEL) is another chaperone system that prevents aggregation of misfolded proteins in an ATP-dependent manner (8). In *E. coli*, Hsp60 (GroEL) binds nonnative protein and sequesters it into the central cavity surrounded by the inside wall of a heptamer ring and capped by Hsp10 (GroES), a co-chaperonin (9). In strain SC2, both Hsp10 and Hsp60 were constitutively expressed at high level under all NH_4_^+^ conditions.

**Potassium metabolism (“salt-in strategy”)**. In strain SC2, high (50 mM and 75 mM) NH_4_^+^ levels significantly induced expression of the K^+^ channel histidine kinase sensor (UniProt ID H7QQ02). This two-component system is involved in sensing and responding to changes in environmental osmolarity. It was already shown decades ago that a sudden osmotic upshock triggers potassium uptake, thereby raising the cytoplasmic K^+^ pool (10). Potassium accumulation is believed to act as an (initial) emergency stress reaction to limit water efflux in high osmolality conditions and, in consequence, to adapt the cell proteins and enzymes to the presence of high extracellular ion concentrations (11, 12). For instance, E. coli possesses four constitutively expressed low affinity K^+^ transport systems [TrkG, TrkH, TrkF, and Kup (formerly TrkD)] and, in addition, a single inducible high affinity ATP-driven K^+^ uptake system, Kdp (13). While Trk is not encoded by the SC2 genome, Kup is constitutively expressed at low level under all NH_4_^+^ conditions. Thus, like in *E. coli* (14), Kup expression is not involved in the osmoregulation in strain SC2. By contrast, the expression level of the entire protein complex (KdpABC) of the high-affinity K^+^ uptake system significantly increased under high (75 mM) NH_4_^+^ load (Table 3). In summary, the significant increase in the expression level of both K^+^ sensing (H7QQ02) and high-affinity uptake (KdpABC) system indicates that strain SC2 operates the “salt in” strategy (15, 16) to cope with the conditions of elevated osmolarity under high NH_4_^+^ load.

**Ammonium assimilation and glutamate/glutamine metabolism.** *Methylocystis* spp. have been reported to assimilate NH_4_^+^ through the GOGAT system, involving glutamine synthetase and glutamine-oxoglutarate amidotransferase (17, 18). Indeed, the intracellular accumulation of glutamate was presumably achieched by ammonia assimilation using the high-affinity GOGAT pathway, given that GOGAT was constitutively expressed at high level under all NH_4_^+^ conditions (Table 3). A high steady-state pool of negatively charged glutamate is presumably employed to neutralize the charge equibilibrium of highly detrimental cation of “salt in” K^+^ in the cell (14, 19, 20) but also to act, in addition to glutamine, as a nitrogen donor for biosynthesis of other amino acids (21-23). Glutamine showed the same intracellular accumulation pattern as did glutamate but on a tenfold lower level (Fig. 4).

Among the canonical amino acids, the intracellular concentrations of threonine and lysine were also significantly increased at high (75 mM) NH_4_^+^ load (Fig. 4). While the exact functional reason for this pool size increase remains elusive, it may point to a supplementary role of amino acids in improving molecular structure and metabolic function of SC2 cells under unfavorable conditions, such as high ionic strength (24).

**Glutathione metabolism.** The functional role of glutathione transferases (GSTs) and glutathione peroxidase (GPX) is to maintain an optimal cytoplasmic redox state, in both bacterial and eukaryotic cells. Following a burst of reactive oxygen species (ROS) or reactive nitrogen species (RNS), GST expression and activity is induced to catalyze the transfer of reactive species to reduced glutathione (GSH), thereby leading to the detoxification of the oxidants and oxidized glutathione (GSSG) (25, 26). In fact, it has been shown that different types of abiotic stress (e.g., osmotic stress, acidity, oxidative stress) trigger elevated cytoplasmic levels of glutathione in bacteria (27), plants (28), and mammals (29). In strain SC2, high NH_4_^+^ load induced a significant increase in the expression level of three isoforms of the GST superfamily and GPX (Table 3). In a parallel experiment, we found that NaCl stress induced the significant upregulation of two GST isoforms with the UniProt IDs J7QAL3 and J7QTI3 (unpublished proteome data). The ionic strength (IS) applied in that experiment was with 153 mM (equal to 0.75% NaCl) even higher than under 50 mM (90 mM IS) and 75 mM (114 mM IS) NH_4_^+^ load. Nonetheless, in addition to GST J7QTI3, the expression of GST isoforms with the UniProt IDs J7QHL1 and J7Q532 were specifically and significantly upregulated in response to increasing NH_4_^+^ load (Table 3). Their increase in expression level may be induced by the increased production of both hydroxylamine (30) and RNS such as nitrite (31, 32), given that the expression of both GST isoforms specifically responded to increasing NH_4_^+^ load. GST J7QAL3 was undetectable under all NH_4_^+^ conditions. High cellular levels of glutathione may also be required for the retention of K^+^ in the initial stage of osmotic adaptation (33).

Notably, the glutathione metabolism pathway has multiple stress-responsive functions in methanotrophs, among these formaldehyde detoxification. A significant upregulation of glutathione (GSH)-dependent formaldehyde detoxification was observed in methanol-grown cultures of *Methylomicrobium album* BG8. During the detoxification response, GSH is condensed with formaldehyde either spontaneously or via formaldehyde-activated enzyme to yield S-hydroxymethylglutathione, which is subsequently converted to formate through a multistep reaction that releases GSH for another round of formaldehyde detoxification (34).

***Differential regulation of proteins encoded on pBSC2-1 and pBSC2-2***

Strain SC2 harbors two large, low-copy-number plasmids of 229.6 kb (pBSC2-1) and 143.5 kb (pBSC2-2) in size (35). Various plasmid-encoded proteins were differentially regulated in response to increasing NH_4_^+^ load: 26 of 53 proteins on pBSC2-1 and 24 of 45 proteins on pBSC2-2, with most of them being significantly upregulated (Table S10).

***pBSC2-1***
In particular, the single-stranded DNA-binding protein (SBB), three-component CzcCBA complex, and subunits of the F_0_F_1_ ATPase complex were among the differentially regulated proteins. The SBB protein showed a gradual increase in the expression level with increasing NH_4_^+^ load. Indeed, SBB was the most significantly upregulated (3.95-fold) plasmid-encoded protein at 75 mM NH_4_^+^ (see Data Set S6 at <https://doi.org/10.6084/m9.figshare.20750215.v2>). The SBB protein plays a major role in DNA replication, recombination, and repair. SSB binds to ssDNA in order to (i) keep the individual strands separated by holding them in place so that each strand can serve as a template for new DNA synthesis, (ii) protect ssDNA from being broken down by nucleases during repair and (iii) remove the secondary structure of ssDNA so that other enzymes are able to access them and act effectively upon the strands (36, 37). In transcriptomics, the *ssb* gene also showed significantly increased expression level when SC2 cells were exposed to 0.75 % NaCl (38). Thus, it is reasonable to conclude that the SBB protein plays an important biological role in the cellular response of strain SC2 to high ionic strength.

The *czcCBA* gene cluster is found on diverse plasmids (39) and encodes three proteins: CzcA (the RND family specific protein), two-subunit CzcB ([membrane](https://en.wikipedia.org/wiki/Membrane_protein) fusion protein, MFP), and CzcC (outer membrane protein, OMF). The three proteins constitute a membrane-bound tripartite complex of the RND family (40). This efflux system has been reported to confer resistance to metal cations, including but not limited to zinc, cadmium, and cobalt (41). Its expression is regulated through metal ions (42). Tripartite RND efflux systems are known to be involved in maintaining cellular homeostasis by removal of toxic compounds (43, 44). All three proteins (CzcA, CzcB, CzcC) were detected in the pBSC2-1 proteome, but the regulation of their expression in response to increasing NH_4_^+^ load differed. While the expression level of CzcA and both subunits of CzcB were found to be significantly upregulated under high (50 mM and 75 mM) NH_4_^+^ levels, CzcC was constitutively expressed at low level under all NH_4_^+^ conditions. It has been shown that CzcA transports metal cations from the cytoplasm to the periplasmic substrate-binding sites, where the outer membrane protein (CzcC) and membrane fusion protein (CzcB) contribute to the periplasmic efflux of the metal ions (43). CzcA, localized in the inner membrane, is active through proton motive force, which is an ATP-dependent process (44).

Both the overall structure and the amino acid sequence homology of ATP synthases are conserved among all organisms. The ATP synthase of *E. coli,* which has been most intensively studied, is composed of eight different subunits; with three subunits (a, b, and c) constituting the membrane-integrated ion-translocating F_0_ complex. The other five subunits (α, β, γ, δ, and ε) belong to the peripheral F_1_ complex. The F_0_F_1_-ATPases of bacteria serve two important physiological functions. The enzyme catalyzes the synthesis of ATP from ADP and inorganic phosphate utilizing the energy of an electrochemical ion gradient. On the other hand, under conditions of low driving force, ATP synthases function as ATPases, thereby generating a transmembrane ion gradient at the expense of ATP hydrolysis (45-47). In our study, all ATPase subunits, including *a*, *b*, *c* subunits of F_0_ complex and α, β, γ, δ, ε subunits of F_1_ complex, are encoded by pBSC2-1 and were detectable in the SC2 proteome at low level under standard growth conditions with 1 mM NH_4_^+^. In particular, the *b* and *c* subunits of F_0_ complex, but also α, β, γ subunits of F_1_ complex, were significantly upregulated in response to high (50 mM and 75 mM) NH_4_^+^ levels. This increase in expression level, concurrently with CzcA, may suggest that the pBSC2-1 encoded F_0_F_1_ ATPase provides the energy for the efflux transporter activity of CzcA.

***pBSC2-2***
In particular, the type IV secretion system (T4SS) was among the differentially regulated proteins. T4SS is a secretion protein complex with versatile functions found in both gram-negative and gram-positive bacteria, and archaea (48-50). Three main functions of T4SS are known: conjugation, DNA exchange with the extracellular space, and delivery of proteins to target cells (50). The T4SS system of many gram-negative bacteria comprises 12 proteins (48, 51), whose encoding genes were all detected on both plasmids, pBSC2-1 and pBSC2-2 (35). According to the UniProt database of *Methylocystis* sp. strain SC2, their designation is VirB1 to VirB11 (pBSC2-1) and AvhB1 to AvhB11 (pBSC2-2). The twelfth T4SS protein differs between the two plasmids and is TraG (pBSC2-1) and VirD4 (pBSC2-2). Nine T4SS proteins (AvhB1, AvhB4, AvhB5, AvhB7, AvhB8, AvhB9, AvhB10, AvhB11, VirD4) were identified in the pBSC2-2 proteome, of which seven T4SS proteins were significantly overexpressed at high (75 mM) NH_4_^+^ load (see Data Set S6 at <https://doi.org/10.6084/m9.figshare.20750215.v2>). Among these are AvhB4, AvhB11, and VirD4, which all participate in the synthesis of ATPase powering pilus assembly. The other four upregulated proteins are the inner (AvhB8, AvhB10) and outer (AvhB7, AvhB9) membrane channel proteins (48, 52).

All the T4SS proteins encoded by pBSC2-1 and pBSC2-2 have a conjugative transfer function (35), but showed an opposite expression pattern when SC2 cells were exposed to either 0.75% NaCl or high NH_4_^+^ load. The pBSC2-1 encoded T4SS proteins were significantly upregulated in response to the incubation treatment with 0.75% NaCl (38), but not under high NH_4_^+^ load (this study). By contrast, the pBSC2-2 encoded T4SS proteins were significantly upregulated in response to high NH_4_^+^ load, but not in the incubation treatment with 0.75% NaCl. These findings suggest that the T4SS systems encoded by either pBSC2-1 or pBSC2-2 differ in their conjugative transfer function. The TraG (pBSC2-1) and VirD4 (pBSC2-2) proteins have the fundamental function to recruit substrates to the T4SS system for secretion through the translocation channel (48), but substrate recognition may differ between TraG and VirD4 (52). This may explain the opposite expression response of the pBSC2-1 and pBSC2-2 encoded T4SS proteins to 075% NaCl and high NH_4_^+^ load.

In summary, proteins located on pBSC2-1 and pBSC2-2 showed a strong differential regulation in response to increasing NH_4_^+^ load, thereby suggesting a molecular cross-talk between the two plasmids and the SC2 chromosome. Notably, previous attempts to cure plasmids pBSC2-1 and pBSC2-2 in strain SC2 failed (35). Both the failure to cure the two plasmids and the stress-induced proteome adjustments may indicate a stability relationship between the SC2 chromosome and the two plasmids, pBSC2-1 and pBSC2-2; not just for surviving, but also for conferring *Methylocystis* sp. strain SC2 with improved ability to cope with environmental stress. This view is further supported by the differential regulation of a single PmoC subunit uniquely encoded on pBSC2-2 (Table 3) and the constitutive expression of a *nos* operon located on the same plasmid (Fig. S9). Indeed, the PmoC subunit showed the greatest downregulation (- 4.8-fold) among all differentially regulated proteins in response to high NH_4_^+^ load.

**References**

1. Haikarainen T, Papageorgiou AC. 2010. Dps-like proteins: structural and functional insights into a versatile protein family. Cell Mol Life Sci 67:341-351.

2. Karas VO, Westerlaken I, Meyer AS, Gourse RL. 2015. The DNA-Binding Protein from Starved Cells (Dps) Utilizes Dual Functions To Defend Cells against Multiple Stresses. J Bacteriol 197:3206-3215.

3. Almirón M, Link AJ, Furlong D, Kolter R. 1992. A novel DNA-binding protein with regulatory and protective roles in starved *Escherichia coli*. Genes Dev 6:2646-54.

4. Nair S, Finkel SE. 2004. Dps protects cells against multiple stresses during stationary phase. J Bacteriol 186:4192-4198.

5. Pragai Z, Harwood CR. 2002. Regulatory interactions between the Pho and sigma(B)-dependent general stress regulons of *Bacillus subtilis*. Microbiology (Reading) 148:1593-1602.

6. Narberhaus F, Balsiger S. 2003. Structure-function studies of *Escherichia coli* RpoH (sigma32) by in vitro linker insertion mutagenesis. J Bacteriol 185:2731-8.

7. Zietkiewicz S, Krzewska J, Liberek K. 2004. Successive and synergistic action of the Hsp70 and Hsp100 chaperones in protein disaggregation. J Biol Chem 279:44376-83.

8. Watanabe YH, Motohashi K, Taguchi H, Yoshida M. 2000. Heat-inactivated proteins managed by DnaKJ-GrpE-ClpB chaperones are released as a chaperonin-recognizable non-native form. J Biol Chem 275:12388-92.

9. Rye HS, Roseman AM, Chen S, Furtak K, Fenton WA, Saibil HR, Horwich AL. 1999. GroEL-GroES cycling: ATP and nonnative polypeptide direct alternation of folding-active rings. Cell 97:325-338.

10. Whatmore AM, Chudek JA, Reed RH. 1990. The Effects of Osmotic Upshock on the Intracellular Solute Pools of *Bacillus-Subtilis*. J Gen Microbiol 136:2527-2535.

11. Gunde-Cimerman N, Plemenitaš A, Oren A. 2018. Strategies of adaptation of microorganisms of the three domains of life to high salt concentrations. FEMS Microbiol Rev 42:353-375.

12. Hoffmann T, Bremer E. 2017. Guardians in a stressful world: the Opu family of compatible solute transporters from *Bacillus subtilis*. Biol Chem 398:193-214.

13. Roe AJ, McLaggan D, O'Byrne CP, Booth IR. 2000. Rapid inactivation of the *Escherichia coli* Kdp K^+^ uptake system by high potassium concentrations. Molecular Microbiology 35:1235-1243.

14. Sleator RD, Hill C. 2002. Bacterial osmoadaptation: the role of osmolytes in bacterial stress and virulence. FEMS Microbiol Rev 26:49-71.

15. Bremer E. 2000. Coping with osmotic challenges: osmoregulation through accumulation and release of compatible solutes in *B. subtilis*. Comparative Biochemistry and Physiology Part A: Molecular & Integrative Physiology 126:17.

16. Oren A. 2013. Life at high salt concentrations, intracellular KCl concentrations, and acidic proteomes. Front Microbiol 4.

17. Mustakhimov II, Rozova ON, Solntseva NP, Khmelenina VN, Reshetnikov AS, Trotsenko YA. 2017. The properties and potential metabolic role of glucokinase in halotolerant obligate methanotroph *Methylomicrobium alcaliphilum* 20Z. Antonie Van Leeuwenhoek 110:375-386.

18. Zuñiga C, Morales M, Revah S. 2013. Polyhydroxyalkanoates accumulation by *Methylobacterium organophilum* CZ-2 during methane degradation using citrate or propionate as cosubstrates. Bioresource Technol 129:686-689.

19. Sadeghi A, Soltani BM, Nekouei MK, Jouzani GS, Mirzaei HH, Sadeghizadeh M. 2014. Diversity of the ectoines biosynthesis genes in the salt tolerant Streptomyces and evidence for inductive effect of ectoines on their accumulation. Microbiol Res 169:699-708.

20. Yan D. 2007. Protection of the glutamate pool concentration in enteric bacteria. PNAS 104:9475-9480.

21. van Heeswijk WC, Westerhoff HV, Boogerd FC. 2013. Nitrogen assimilation in *Escherichia coli*: putting molecular data into a systems perspective. Microbiol Mol Biol Rev 77:628-695.

22. Liu G, Vijayaraman SB, Dong Y, Li X, Andongmaa BT, Zhao L, Tu J, He J, Lin L. 2020. *Bacillus velezensis* LG37: transcriptome profiling and functional verification of GlnK and MnrA in ammonia assimilation. BMC Genomics 21:215.

23. Viljoen AJ, Kirsten CJ, Baker B, van Helden PD, Wiid IJ. 2013. The role of glutamine oxoglutarate aminotransferase and glutamate dehydrogenase in nitrogen metabolism in *Mycobacterium bovis* BCG. Plos One 8:e84452.

24. Zaprasis A, Bleisteiner M, Kerres A, Hoffmann T, Bremer E. 2015. Uptake of Amino Acids and Their Metabolic Conversion into the Compatible Solute Proline Confers Osmoprotection to *Bacillus subtilis*. Appl Environ Microb 81:250-259.

25. Allocati N, Federici L, Masulli M, Di Ilio C. 2009. Glutathione transferases in bacteria. FEBS J 276:58-75.

26. Allocati N, Federici L, Masulli M, Di Ilio C. 2012. Distribution of glutathione transferases in Gram-positive bacteria and Archaea. Biochimie 94:588-596.

27. Smirnova GV, Krasnykh TA, Oktyabrsky ON. 2001. Role of Glutathione in the Response of *Escherichia coli* to Osmotic Stress. Biochemistry (Moscow) 66:973-978.

28. Yang S, Hao D, Jin M, Li Y, Liu Z, Huang Y, Chen T, Su Y. 2020. Internal ammonium excess induces ROS-mediated reactions and causes carbon scarcity in rice. BMC Plant Biology 20:143.

29. Al-Fageeh MB, Smales CM. 2006. Control and regulation of the cellular responses to cold shock: the responses in yeast and mammalian systems. Biochem J 397:247-259.

30. Spooren AA, Evelo CT. 1998. Only the glutathione dependent antioxidant enzymes are inhibited by haematotoxic hydroxylamines. Human & Experimental Toxicology 17:554-559.

31. Stein LY, Klotz MG. 2011. Nitrifying and denitrifying pathways of methanotrophic bacteria. Biochem Soc Trans 39:1826-31.

32. Tharmalingam S, Alhasawi A, Appanna VP, Lemire J, Appanna VD. 2017. Reactive nitrogen species (RNS)-resistant microbes: adaptation and medical implications. Biol Chem 398:1193-1208.

33. Smirnova GV, Oktyabrsky ON. 2005. Glutathione in Bacteria. Biochemistry (Moscow) 70:1199-1211.

34. Sugden S, Lazic M, Sauvageau D, Stein LY. 2022. Transcriptomic and metabolomic responses to carbon and nitrogen sources in *Methylomicrobium album* BG8. Appl Environ Microbiol 87:e00385-21.

35. Dam B, Kube M, Dam S, Reinhardt R, Liesack W. 2012. Complete Sequence Analysis of Two Methanotroph-Specific repABC-Containing Plasmids from *Methylocystis* sp. Strain SC2. Appl Environ Microb 78:4373-4379.

36. Antony E, Lohman TM. 2019. Dynamics of E. coli single stranded DNA binding (SSB) protein-DNA complexes. Semin Cell Dev Biol 86:102-111.

37. Kunzelmann S, Morris C, Chavda AP, Eccleston JF, Webb MR. 2010. Mechanism of Interaction between Single-Stranded DNA Binding Protein and DNA. Biochemistry-Us 49:843-852.

38. Han DF, Link H, Liesack W. 2017. Response of *Methylocystis* sp Strain SC2 to Salt Stress: Physiology, Global Transcriptome, and Amino Acid Profiles. Appl Environ Microb 83.

39. Niu X-N, Wei Z-Q, Zou H-F, Xie G-G, Wu F, Li K-J, Jiang W, Tang J-L, He Y-Q. 2015. Complete sequence and detailed analysis of the first indigenous plasmid from *Xanthomonas oryzae* pv. *oryzicola*. BMC Microbiology 15:233.

40. Hoffmann F, Rinas U. 2004. Stress Induced by Recombinant Protein Production in *Escherichia coli*, p 73-92, Physiological Stress Responses in Bioprocesses: -/- doi:10.1007/b93994. Springer Berlin Heidelberg, Berlin, Heidelberg.

41. Valencia EY, Braz VS, Guzzo C, Marques MV. 2013. Two RND proteins involved in heavy metal efflux in *Caulobacter crescentus* belong to separate clusters within proteobacteria. BMC Microbiology 13:79.

42. Nies DH. 2003. Efflux-mediated heavy metal resistance in prokaryotes. FEMS Microbiol Rev 27:313-339.

43. Kim EH, Nies DH, McEvoy MM, Rensing C. 2011. Switch or funnel: how RND-type transport systems control periplasmic metal homeostasis. J Bacteriol 193:2381-7.

44. Issa KHB, Phan G, Broutin I. 2018. Functional Mechanism of the Efflux Pumps Transcription Regulators From *Pseudomonas aeruginosa* Based on 3D Structures. Front Mol Biosci 5.

45. Cabezón E, Montgomery MG, Leslie AGW, Walker JE. 2003. The structure of bovine F1-ATPase in complex with its regulatory protein IF1. Nat Struct Mol Biol 10:744-750.

46. Guo H, Suzuki T, Rubinstein JL. 2019. Structure of a bacterial ATP synthase. Elife 8.

47. Deckers-Hebestreit G, Altendorf K. 1996. The F0F1-type ATP synthases of bacteria: structure and function of the F0 complex. Annu Rev Microbiol 50:791-824.

48. Fronzes R, Christie PJ, Waksman G. 2009. The structural biology of type IV secretion systems. Nat Rev Microbiol 7:703-14.

49. Christie PJ, Whitaker N, González-Rivera C. 2014. Mechanism and structure of the bacterial type IV secretion systems. Biochimica et Biophysica Acta (BBA) - Molecular Cell Research 1843:1578-1591.

50. Wallden K, Rivera-Calzada A, Waksman G. 2010. Type IV secretion systems: versatility and diversity in function. Cell Microbiol 12:1203-12.

51. Costa TRD, Felisberto-Rodrigues C, Meir A, Prevost MS, Redzej A, Trokter M, Waksman G. 2015. Secretion systems in Gram-negative bacteria: structural and mechanistic insights. Nat Rev Microbiol 13:343-359.

52. Christie PJ. 2004. Type IV secretion: the Agrobacterium VirB/D4 and related conjugation systems. Biochim Biophys Acta 1694:219-234.
